# *Bacillus megaterium* adapts to acid stress condition through a network of genes: Insight from a genome-wide transcriptome analysis

**DOI:** 10.1038/s41598-018-34221-0

**Published:** 2018-10-31

**Authors:** Gunajit Goswami, Debashis Panda, Ramkrishna Samanta, Robin Chandra Boro, Mahendra Kumar Modi, Kamal Malla Bujarbaruah, Madhumita Barooah

**Affiliations:** 1 Department of Agricultural Biotechnology, Assam Agricultural University, Jorhat, 785013 India; 2 Department of Life-Sciences, Dibrugarh University, Dibrugarh, 786004 Assam India; 3 Distributed Information Centre, Department of Agricultural Biotechnology, Assam Agricultural University, Jorhat, 785013 India

## Abstract

RNA-seq analysis of *B*. *megaterium* exposed to pH 7.0 and pH 4.5 showed differential expression of 207 genes related to several processes. Among the 207 genes, 11 genes displayed increased transcription exclusively in pH 4.5. Exposure to pH 4.5 induced the expression of genes related to maintenance of cell integrity, pH homeostasis, alternative energy generation and modification of metabolic processes. Metabolic processes like pentose phosphate pathway, fatty acid biosynthesis, cysteine and methionine metabolism and synthesis of arginine and proline were remodeled during acid stress. Genes associated with oxidative stress and osmotic stress were up-regulated at pH 4.5 indicating a link between acid stress and other stresses. Acid stress also induced expression of genes that encoded general stress-responsive proteins as well as several hypothetical proteins. Our study indicates that a network of genes aid *B*. *megaterium* G18 to adapt and survive in acid stress condition.

## Introduction

Bacteria have innate ability to survive and grow in several stress conditions including extreme ecological niches. Acidic stress condition specifically, soil acidic condition has significant relevance and ramification in agriculture, food, and human health. The importance of acid stress has propelled several studies in unraveling the molecular mechanisms of acid tolerance in bacteria especially the pathogenic enteric bacteria and those involved with food and beverages.

Neutralophilic bacteria adopt a number of mechanisms in maintaining pH homeostasis including no growth strategy as adopted by *Escherichia coli* and *Salmonella* spp., to survive during exposure to pH values that are outside their growth range^1,2^. Survival and growth under stress involve changes in cell structure, metabolism, and transport patterns (F1F0-ATPase, ABC transporter). The changes in membrane fatty acid profiles in response to dropping in pH point out the significant role of the cell membrane in acid tolerance^3^. The role of amino acid decarboxylases in maintaining bacterial pH homeostasis has also been reported^4^. Examples of these systems include lysine, arginine, and glutamate decarboxylases that combine a proton with internalized amino acid (lysine, arginine, or glutamate) and exchange the product for another amino acid^5^. Protection or repair of macromolecules such as DNA and proteins as essential acid resistance mechanism has also been described by several researchers^3,6,7^. Generation of alkali more specifically, ammonia using the urease and arginine deiminase (ADI) pathways is yet another mechanism adopted by bacteria to tide over acid stress. The acid resistance in gram-positive bacteria is also affected by cell density, as higher cell densities aid in cell to cell communication and formation of biofilm^8^.

*B*. *megaterium* is a Gram-positive, aerobic spore-forming neutralophilic bacterium found in diverse habitats but commonly regarded as a soil bacterium. Its ability to utilize different carbon sources and grow at a wide temperature range (3 °C to 45 °C) makes it an ideal industrial organism^9,10^. *B*. *megaterium* possesses plant growth promoting activity including biocontrol ability against plant pathogens^11,12^. Over the past decade, several studies relating to the genetics of *B*. *megaterium* have been reported and a number of stains have been developed and characterized for several features including auxotrophy, recombination, division, sporulation, germination, antibiotic resistance, UV sensitivity, and neutral protease^13^. However, the molecular mechanisms underlying the ability of *B*. *megaterium* to withstand stress condition such as acid stress has not been addressed. We previously reported the predominance of the genus *Bacillus* in acidic soil condition of Assam based on culture-dependent assay and isolation of an isolate of *B*. *megaterium* G18 that could grow at pH 4.5^14^. This isolate was used in the present study to decipher the underlying mechanism of acid tolerance through a differential transcriptome profiling.

In this paper, we report the transcriptome profile of *B*. *megaterium* G18 subjected to acid stress condition (pH 4.5) and neutral condition (pH 7.0) using the next generation sequencing technology. Next-generation sequencing technology (RNA-Seq) is being used increasingly to analyze the transcriptome profiles during exposure to different stress conditions^15–19^. The role of some of the differentially expressed genes was further validated through qPCR. To the best of our knowledge, this is the first differential transcriptomics analysis of the response of *B*. *megaterium* G18 to an acidic stress condition.

## Results

### Acid shift growth curve and acid tolerance response

**Figure 1:**
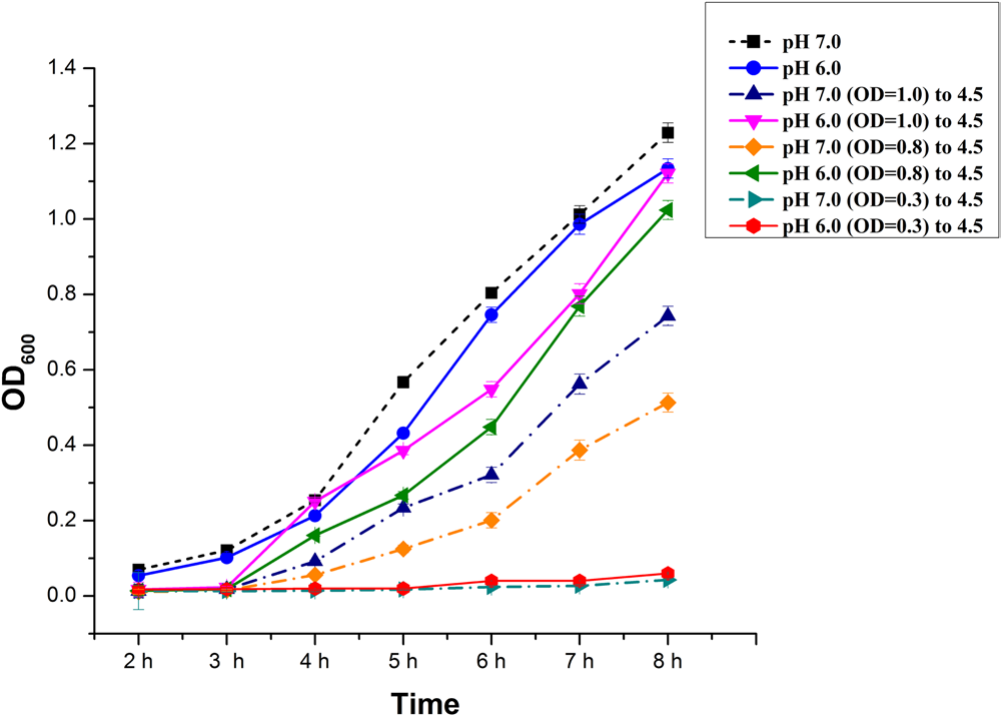
Acid shift growth curve of *B*. *megaterium* G18. Acid and base shift growth curves. *B*. *megaterium* G18 cultures grown overnight in NB were diluted 50-fold and grown at 37 °C in NB adjusted to pH 6.0 and pH 7.0 until an OD_600_ of 0.3, 0.8 and 1.0. The culture at pH 6.0 and pH 7.0 was then diluted 50-fold in NB at pH 4.5 and the diluted cultures were incubated at 37 °C. The average of three biological replicates is shown for each condition. Error bars represent SEM.

*B*. *megaterium* G18 was tested for pH-dependent induction of acid resistance. When the cells grown on pH 7.0 and pH 6.0 of different cell densities were shifted to pH 4.5, a lag of 3 h was observed after which the cells entered the exponential phase. However, the cells grown at pH 6.0 had shorter lag phase than the cells grown at pH 7.0. When the cells grown to early log phase (OD_600_ of 0.3) at pH 7.0 and 6.0 were shifted to pH 4.5, growth was negligible (Fig. 1). This indicated that early log phase cells of *B*. *megaterium* G18 were more susceptible to extreme pH than the late log phase cells. Cells grown at pH 6.0 till OD_600_ of 1.0, 0.8 and 0.3 showed 90 ± 2%, 80 ± 5%, and 45 ± 5% survival respectively after 4 h of exposure at pH 4.5 which decreased to 80 ± 2%, 65 ± 5% and 35 ± 5% respectively when grown at pH 7.0 till OD_600_ of 1, 0.8 and 0.3. This indicated that pre-exposure of *B*. *megaterium* G18 cells to moderate acid induced mechanisms that increase the rate of survival in extreme acid. Although there was no change in the pH of the medium till 4 h of incubation, it gradually increased from 4.5 to 5.8 till 8 h and to 6.68 after an overnight incubation.

### Illumina draft reads

Sequencing of *B. megaterium* cDNA samples, grown at pH 7.0 and pH 4.5 yielded a total of 10,504,233 and 10,505,911 high-quality reads after preprocessing. The clean reads were aligned to the reference genome and 73.58% (control) and 66.42% (acid stress) mapping rates were obtained (Supplementary Table S2). The mapping rate of the sequence reads obtained in this study are similar to those obtained in other studies^16,20^.

### Differential genes expression profile, Cluster analysis, and Functional annotation

**Figure 2:**
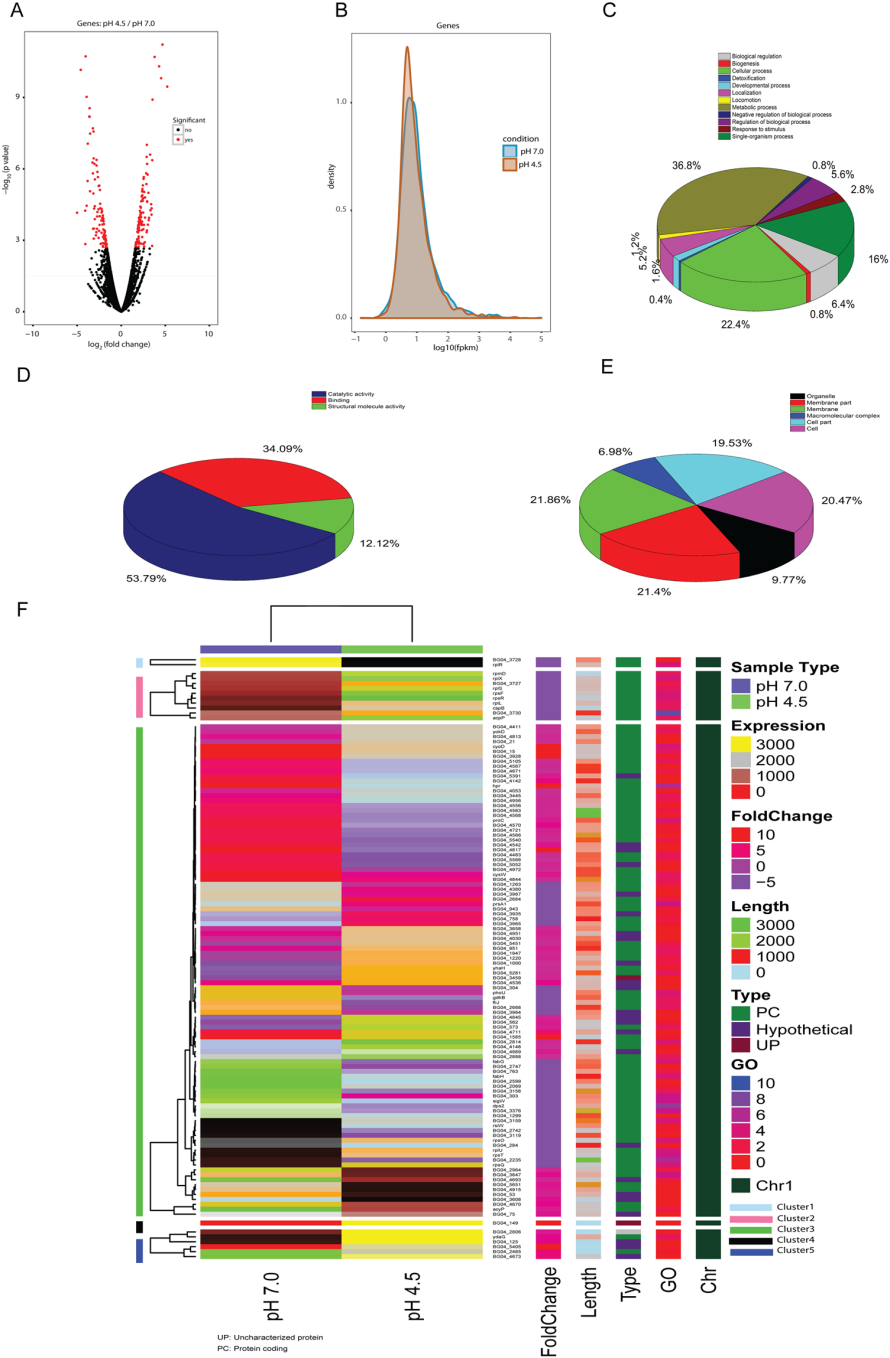
(**A**) Volcano plots showing the overall scatter of gene transcription of *B*. *megaterium* G18. (P-value < 0.01 and log2 fold-change ≥±2 were considered as significantly regulated (Black dots: non-significant genes, Red dots: significant genes). (**B**) Distribution of gene expression level between the control and acid-stressed sample; (**C**–**E**) Functional categories of the differentially expressed genes, broadly grouped into ‘biological process’, ‘cellular component’, and ‘molecular function’ based on Gene Ontology. (**F**) Heatmap of log_2_ fold-change value of differently expressed genes in *B*. *megaterium* G18 during acid stress. (Color code for different classes: Purple: pH 7.0 and Green: pH 4.5).

A comparison of the transcripts between control (pH 7.0) and acid-stressed (pH 4.5) sample revealed 207 genes to be differentially expressed (Fig. 2A,B**)**. The GO assignments (Biological Process, Molecular Function, and Cellular Component) were used to classify the functions of the differentially expressed *B*. *megaterium* genes under acid stress. Based on sequence homology, 207 transcripts were assigned with at least one GO term at the second level (Fig. 2C–E). A total of 108 genes were found to be associated with biological processes, 98 genes were involved in performing molecular functions and rest 82 genes were related to cellular components, however, some of the genes were common to all. The functions of the differentially expressed genes were related to cell wall remodeling and sporulation, carbohydrate metabolism, protein metabolism, fatty acid metabolism, stress response, motility, amino acid biosynthesis, amino acid decarboxylation, regulation of transcription, transport of ions and molecules, energy generation, and some genes with unknown function(s). Genes relating to maintenance of cell integrity (membrane integrity) which showed increase in expression under acid stress include BG04_3445 (encoding capsular biosynthesis protein), BG04_4142 (encoding cupin family protein), BG04_5651 (encodes penicillin binding protein) and BG04_4289 (encodes polysaccharide deacetylase).

Genes relating to sporulation includes BG04_2465 (encoding Spo0A-P phosphatase or SpoE like protein) and BG04_3658 (encoding sporulation family protein) also showed differential expression under acid stress.

Among the different genes involved in carbohydrate metabolism, genes encoding L-lactate dehydrogenase (BG04_2814) and acylphosphatase (*acyP*) showed up-regulation at pH 4.5. Some of the genes related to alternative energy generation such as formate dehydrogenase (BG04_4915), cytochrome O ubiquinol oxidase IV (*cyoD*) showed an increase in expression under acid stress. Similar to other bacteria^21–23^, acid stress also induced the expression of genes that are related to pH homeostasis and stress response. The genes that code for glutamate decarboxylase, protease, peptidase and general stress protein showed increase in transcription at pH 4.5. Genes encoding transcriptional regulators such as Hpr (*hpr*), crp/fnr (BG04_2965) and rrf2 family transcriptional regulator (BG04_20) and other transcriptional regulator showed differential expression in acid stress. The gene BG04_15 that codes for family transcriptional regulator showed exclusive up-regulation at pH 4.5. Increased expression of *proC* gene that codes for pyrroline-5-carboxylate reductase involved in proline biosynthesis was observed in acid stress. The transcriptome data also showed an increased expression of genes encoding proteins related to iron and sulfur metabolisms which include bacterioferritin (BG04_3847), GNAT family n-acetyltransferases (BG04_4956), heme-degrading monooxygenase (BG04_2888), Fe-S cluster biogenesis protein (BG04_3482), cysteine desulfurase (BG04_5281) and sulfate ABC transporter permease subunit (*cysW*).

Increasing the stringency by changing the log_2_fold threshold to ≥ ±2 and decreasing FDR threshold to <0.01 reduced the count of differentially expressed genes to 119 genes. Out of these 119 DEGs, 62 genes were up-regulated; 49 genes were down-regulated and 8 genes showed exclusive up-regulation in pH 4.5 (Supplementary Table S3). The cluster analysis of the DEGs revealed 5 clusters of which cluster 1 and 2 contained only down-regulated genes, cluster 4 and 5 contained only up-regulated genes and cluster 3, which was far larger than all other clusters, contained both up-regulated and down-regulated genes (Fig. 2F).The network analysis showed interrelation among DEGs and GO-terms associated with different pathways (Supplementary Fig. S1).

Kyoto Encyclopaedia of Genes and Genomes (KEGG) enrichment analysis^24–26^ revealed that the DEGs between the control and acid stress groups were significantly involved in the Arginine and Proline metabolism pathway (Supplementary Fig. S2), Pentose Phosphate pathway (Supplementary Fig. S3), Vitamin B6 metabolism (Supplementary Fig. S4), and Cysteine and Methionine metabolism (Supplementary Fig. S5).

### Verification of transcriptome data by real-time RT-PCR

**Figure 3:**
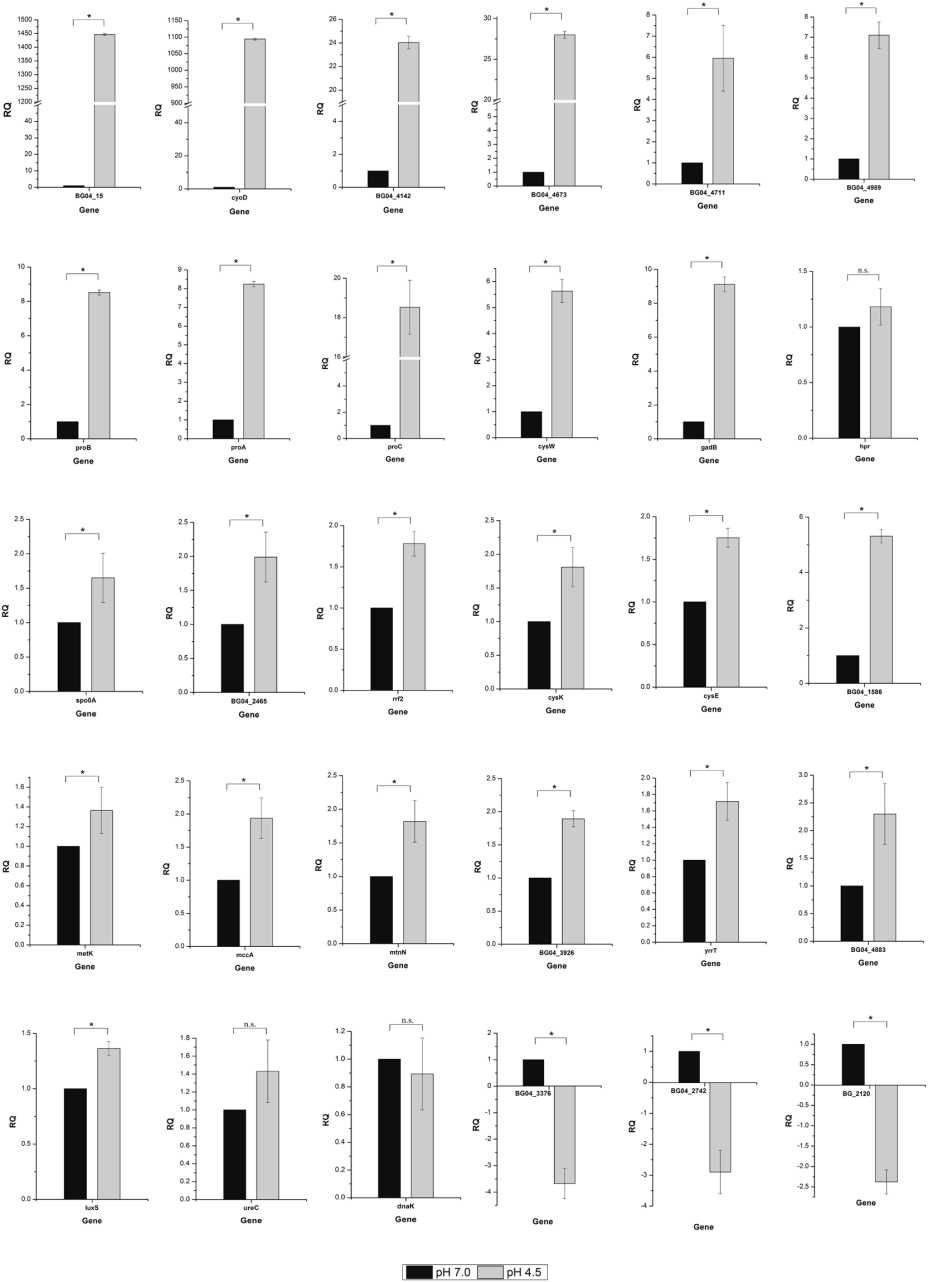
Validation of RNAseq results by measuring the relative expression level of 30 differentially expressed genes in *B. megaterium* G18 by qRT-PCR. Error bar indicates standard error of the mean (n = 3). The *symbol represents significant difference and n.s. represents the non-significant difference.

The reverse transcription products of 31 genes (15 up-regulated and 5 down-regulated in acid stress; 10 not flagged in the transcriptomic study and 1 as an internal standard) were measured by real-time RT-PCR after acid shock treatment for 4 h. Representative acid-responsive genes selected on the basis of their expression pattern in transcriptome analysis showed similar expression pattern in the qPCR analysis (Fig. 3). Relative expression levels of the genes that are not flagged in the transcriptomic study but either reported to be acid-induced (*ureC* and *dnaK*) or sharing the same operon with upregulated gene *pro*C *viz*. *proA*, *proB*, were also measured. The transcripts of both *proA* and *proB* were increased in response to low pH; on the contrary *ureC* and *dnaK* did not show significant differences in their expression in acid stress. The qPCR result of *ureC* confirmed the result of urease test (Supplementary Fig. S6). The qPCR analysis of the gene BG04_20 and the genes related to cysteine biosynthesis (Supplementary Table S1) pathway showed upregulation under acid stress. The genes that regulate sporulation (*spo0A* and BG04_2465) also showed increased expression under acid stress. However, no significant difference was observed in the expression of *hpr* under acid stress.

## Discussion

Low pH in the environment is known to influence bacterial growth characteristics; however, they are able to adapt to environmental stress conditions including acid stress through deployment of several mechanisms. In the present work, we investigated the physiological and molecular responses of *B*. *megateirum* G18 to pH 4.5. A pre-exposure to pH 6.0, before exposing the cells of *B*. *megaterium* G18 to acidic condition (pH 4.5) considerably reduced the duration of lag phase. In addition, the ability of *B*. *megaterium* to tolerate low pH was also dependent upon cell density as early log phase cultures were more susceptible to low pH than the late log phase cultures (Fig. 1). In *B*. *subtilis* and other bacterial species, pre-exposure to moderate acid enable the cells to survive under extreme acidic condition by a process known as acid tolerance response (ATR response)^2,27–29^.

Neutralophilic bacteria employ several mechanisms to survive in acid stress^22,23^. One of the basic strategies is the maintenance of the cell wall/membrane integrity^5^. The cell wall made up of peptidoglycan (PG) protects the cell against lysis due to the osmotic pressure and maintains cell shape. Proteins involved in synthesis, remodeling, and turnover of peptidoglycans play important role in cell growth and division^30^. The penicillin-binding proteins (PBPs) polymerize and modify PG to build the morphology of the peptidoglycan exoskeleton together with cytoskeleton proteins that regulate septum formation and cell shape^31^. Polysaccharide deacetylase has been demonstrated to be associated with lateral PG synthesis, biogenesis of PG during cell division and elongation, neutral polysaccharide attachment to PG as well as polysaccharide modification^32^ that altogether help stabilize the cell wall and aids in adaptation to high salt stress in *B*. *anthracis*^33^. Polysaccharide deacetylase is also associated with sporulation and germination in *B*. *subtilis*^34^. The cupin superfamily of proteins, named on the basis of a conserved β-barrel fold has different functional classes among which, single-domain bacterial enzymes such as isomerases and epimerases are reported to be involved in the modification of cell wall carbohydrates^35^. The GNAT family N-acetyltransferases are reported to be involved in peptidoglycan recycling^36^, detoxification pathways^37,38^, iron acquisition^39,40^ and maintenance of redox balance^41^. The peptidoglycan is composed of alternating N-acetylglucosamine and N-acetylmuramic acid on which the pentapeptide [L-Ala)-(D-Glu)-X-(D-Ala)-(D-Ala] is linked via a lactyl group^42^. In organisms containing the Fem enzymes (a member of gnat family n-acetyltransferases), the pentapeptide is further modified by the addition of up to five amino acids^43^. The fact that several genes that encode membrane proteins and proteins involved in peptidoglycan assembly showed differential transcription (Supplementary Table S3) in our study indicating that *B*. *megaterium* G18 may remodel its membrane structure for maintaining membrane integrity under acid stress.

Spore and biofilm formation is associated with the activation of Spo0A^44^ that aid bacteria to tide over different stress conditons^5^. Although RNAseq data did not indicate upregulation of *spo0A*, qPCR analysis (Fig. 3) showed 1.6 folds increase in transcription of *spo0A* gene under acid stress. Spore formation in *B*. *subtilis* is governed by a master regulatory protein known as Spo0A^45^ which is activated at the start of sporulation by a multicomponent phosphorelay consisting of histidine autokinases and Spo0Fand Spo0B protein. The kinases phosphorylate Spo0F, Spo0F~P subsequently transfers the phosphoryl group to Spo0B (Spo0B~P) which finally transfers the phosphoryl group to Spo0A resulting in activation of Spo0A (Spo0A~P). However, the level of Spo0A~P is also controlled by phosphatases (e.g. RapA and SpoE) that remove phosphoryl groups from Spo0F~P and Spo0A~P resulting in inhibition of sporulation due to lower levels of Spo0A~P^46^. This pathway may also operate in *B*. *megaterium* as per the KEGG pathway map (map02020, Supplementary Fig. S6). Our analysis showed an increase in the expression of the gene encoding Spo0A~P phosphatase (Spo0E family sporulation regulatory protein-aspartic acid phosphatase) that negatively regulates sporulation^47^ indicating spore and biofilm formation was prevented under acid stress which are in accordance with the findings of our microscopic study (Supplementary Fig. S7) and *in vitro* biofilm formation test (Supplementary Fig. S8). However, this data is insufficient to convincingly claim the role of Spo0E phosphatase in inhibiting sporulation and further studies will be required to ascertain its exclusive involvement in this process under acidic stress.

Increased expression of several genes that encode protease and peptidase was observed in our transcriptome data. In stress condition, proteins that undergo denaturation regain their original conformation with the aid of molecular chaperones. Proteins that cannot be re-natured by molecular chaperones may be degraded in order to recycle amino acids for *de novo* protein synthesis^48^. Since, the RNAseq study did not reveal upregulation of the genes encoding molecular chaperons under acid stress, denaturation of proteins due to the stress may be the reason why several protease-encoding genes showed increased expression during acid stress in our case.

Production of ammonia is another mechanism employed by bacteria to survive under acid stress^5^. The expression of genes that code for enzymes involved in ammonia production was unaltered in the present study under acid stress which is further supported by qPCR analysis (Fig. 3**)** as well as biochemical test (Supplementary Figs S9 and S10), suggesting that this pathway may not have role in imparting acid tolerance in *B*. *megaterium* G18.

The unchanged expression of various amino acid transporters and down-regulation of several genes encoding ribosome-associated proteins, proteins involved in translation process as well as some of the acetyltransferases during exposure to low pH may be an indication of interruption of protein synthesis during acid stress unlike other studies^48–50^. The αN-acetylation of proteins is a relatively rare post-translational modification in prokaryotes^51^; however, αN-acetylation of some ribosomal proteins is required for stabilizing the ribosomal stalk complex^52^.

As shown in Supplementary Table S3, some genes encoding bacterioferritin and heme-degrading monooxygenase were highly transcribed during acid stress and which could be related to iron bioavailability and metabolism^53–56^. Iron is an essential cofactor for the function of several enzymes that take part in alternative energetic pathways^48^. In this context, up-regulation of genes encoding formate dehydrogenase, lactate dehydrogenase, cysteine desulfurase, GNAT-family N-acetyltransferases, Fe-S cluster biosynthesis family protein, molybdenum ABC transporter ATP-binding protein, sulfate ABC transporter permease subunit under acid stress may be interrelated to iron availability and could be an alternative route for energy generation. Bacterioferritin and GNAT family N-acetyltransferases are reported to be involved in Fe acquisition^39,53^. Cysteine desulfurases catalyze the desulfuration of L-cysteine to yield L-alanine and free sulfur in order to provide sulfur for the biosynthesis of cofactors and iron-sulfur clusters^57^. Formate dehydrogenase (EC 1.2.1.2, FDH) catalyzes the oxidation of formate to carbon dioxide coupled to reduction of NAD+ into NADH that is subsequently used for ATP synthesis^58^. Fe-S clusters and Mo are the integral component of formate dehydrogenase (FDH) and required during electron transfer. Molybdenum guanine dinucleotide (MGD) is present in enzyme such as formate dehydrogenase (FdhA) and nitrate reductase (NapA) in *E*. *coli*^59,60^. When the cell is under anaerobic or oxygen-limiting condition FDH provide the alternative source of energy. Similarly, L- lactate dehydrogenase also plays important role in providing an alternate substrate for generating energy. In both cases, NAD is reduced to NADH that may be subsequently used for ATP synthesis. The link between acid stress, oxidative stress, and free iron has earlier been reported^61^. Low pH results in elevated iron-mediated lipid peroxidation^62^ as well as the generation of hydroxyl radicals (•OH) due to the reaction of free iron and H_2_O_2_^63,64^. Regulation of free Fe^2+^ is therefore vital for maintaining cellular activities. Iron storage proteins (bacterioferritin) store iron in an inactive form to prevent the formation of harmful hydroxyl radicals and indirectly contribute to diminishing oxidative stress while ensuring the availability of enough iron for metabolic processes^65^. The up-regulation of the gene that encodes bacterioferritin in *B*. *megaterium* G18 under acid stress is likely to be the result of iron released upon acid stress.

An increased expression of the gene (BG04_20) that encodes the Rrf2 family of transcription factor was observed under acid stress in this study. The Rrf2 family of transcription factors controls various metabolic processes including FeS cluster biogenesis (IscR)^66^, nitric oxide detoxification (NsrR)^67^, iron uptake^67^ and cysteine metabolism (CymR)^68^. In *E*. *coli*, Rrf2 family transcription factor participate in Fe-S cluster assembly^69^ and sulfur metabolism^70^. In *B*. *subtilis*, Rrf2 family transcription factor regulates (CymR) cysteine metabolism and cystine uptake^71^. Although, our RNAseq analysis did not reveal significant differential expression of genes involved in cysteine biosynthesis, qPCR analysis of the BG04_20 gene and the genes involved in cysteine biosynthesis showed upregulation under acid stress (Fig. 3). But multiple sequence alignment of amino acid sequences of CymR, IscR and NsrR showed that BG04_20 had higher similarity with IscR of *B*. *thuringensis*, *B*. *mycoides* and *B*. *amyloliquefaciens* (Supplementary Fig. S11). Based on these data, it appears that the Rrf2 family transcription factor of *B*. *megaterium* G18 may be an IscR. However, further studies will be required to ascertain the pathway it regulates under acid stress.

The role of amino acid decarboxylases in helping several bacteria to survive acid stress condition has been demonstrated^5^. The up-regulation of the gene encoding glutamate decarboxylase during acid stress in the present study implicates the involvement of intracellular pH homeostasis mechanism under acid stress in *B*. *megaterium* G18. The glutamate decarboxylase (GAD) system has been reported to be involved in acid tolerance in several bacterial genera. Glutamate decarboxylase helps in intracellular pH homeostasis by consuming protons with glutamate in a decarboxylation reaction that produces gamma-aminobutyrate (GABA) from glutamate. An antiporter system is usually present to couple the uptake of glutamate to the efflux of GABA^5^. However, it was found that the role of GAD in the acid resistance of *B*. *cereus* ATCC 10987 was limited^23^.

The significant up-regulation of the two general stress proteins (*ydaG* and BG04_220) in this study indicated the stress induced by low pH. Other bacteria including *B*. *subtilis* respond to different stress conditions (acid, heat, ethanol and salt stress, as well as energy starvation) by induction of general stress proteins^72,73^. In *B*. *cereus* these general stress proteins have been reported to be involved in cross-protection to various stress conditions^74^.

The present transcriptomic analysis demonstrated an exclusive increase in transcription of gene encoding cytochrome o ubiquinol oxidase subunit IV under acid stress; in agreement with the previous evidence on the role of cytochrome oxidase in stress condition^75–77^. It has been demonstrated that alternative components of the Electron Transport Chain (ETC) are associated with mainly lethal levels of acid shocks^78,79^. Cytochrome *bd* oxidase (CydAB) has been proposed to function in an alternative electron transport chain together with NAD(P)H-dependant dehydrogenases, such as lactate (*ldh*) and alcohol dehydrogenase (*adhA*) showed increased in expression upon exposure of *B*. *subtilis* cells to 15 mM acetic acid (pH 4.5)^80^.

Maintenance of a reducing environment may be another strategy adopted by bacteria to survive under acid stress. We observed an increased transcription of the gene (BG04_4813) encoding thiol-disulfide oxidoreductases that participate in redox reactions and involve in maintaining the reducing environment of the cytoplasm. Thiol-disulfide oxidoreductases catalyze the formation of disulfide bonds in proteins transported across the membrane^81^; disulfide bond isomerization reactions^82^ and reduction reactions during the assembly of cytochrome aa3^83^. In *B*. *subtilis*, member of thiol-disulfide oxidoreductases helps in spore cortex and cytochrome c synthesis^84,85^.

Up-regulation of the gene encoding Crp/Fnr family transcription regulators were observed under acid stress in *B*. *megaterium*. Members of the Crp/Fnr family transcription regulators typically function as transcriptional activators that are involved in responses to a variety of intracellular or extracellular signals, such as anoxia, carbon monoxide, temperature, nitric oxide, and oxidative and nitrosative stress^86–89^.

A relation between ATR and osmotic stress responses was observed in this study. Acid stress increased the transcript level of the genes *proA*, *proB* and *proC* encoding the enzymes of proline biosynthesis pathway which is further supported by the intracellular proline assay (Supplementary Fig. S12). It has been demonstrated that glutamate and/or proline are among several compatible solutes that are synthesized to counteract osmotic, and chaotropic stresses in several bacteria including *B*. *subtilis*^90,91^. Previous reports indicated that *B*. *subtilis* directly acquires exogenously provided glycine betaine and proline for cellular thermo-protection^92^.

Increased expression of the gene that encodes acyl-phosphatase was observed under acid stress. In *E*. *coli* the *yccX* gene that encodes an acyl-phosphatase is significantly up-regulated during manganese oxidation^18^. Although the physiological function of this enzyme not known in great detail, it is reported to be involved in regulation of ion transport across the membrane as well as regulation of glycolysis during oxidative stress. In some bacteria of the genera *Bacillus*, *Streptococcus* and *Clostridium*, induction of acylphosphatase activity leads to rapid increase in pyruvate content^93^. The pyruvate produced is used for biosynthetic reactions^93^ and acts as an antioxidant^94^.

**Figure 4:**
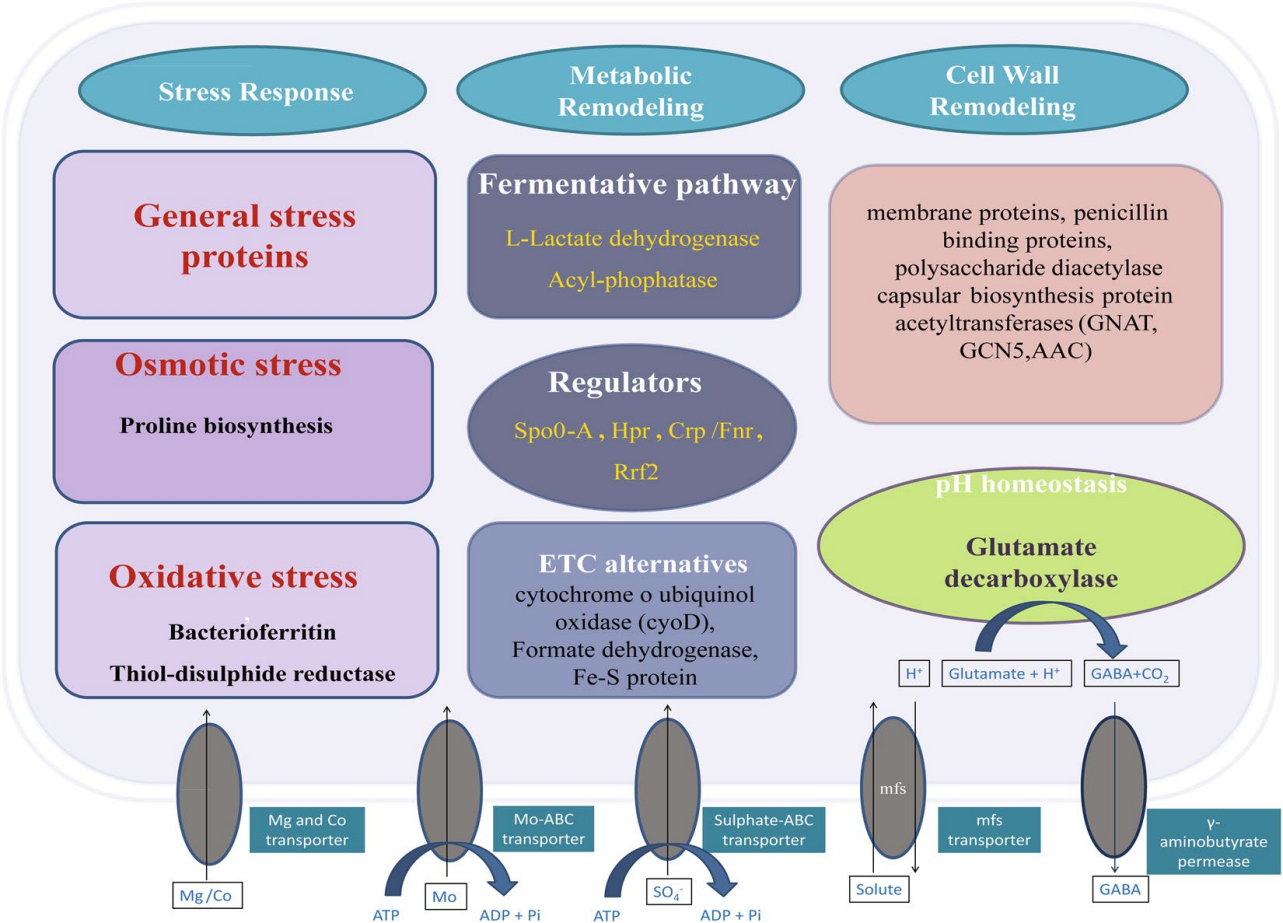
Schematic representation of general acid stress-associated mechanisms in *B*. *megaterium* G18. Several transcriptional regulators, such as Hpr, Crp/Fnr, Rrf2 was changed upon low pH exposure. The metabolic rearrangements observed upon exposure to acid stress were fermentative pathways, such as lactate dehydrogenases (Ldh), and switching glycolysis to pyruvate metabolism (acylphosphatase). pH homeostasis by glutamate decarboxylase that mediates intracellular proton consumption was induced by low pH exposure. The oxidative response may not be directly involved in the resistance to acid stress; however, genes involved in oxidative stress were shown to be upregulated upon exposure to low pH. The electron transfer chain (ETC) is conceivably disturbed by low pH, generating superoxide. Superoxide can lead to the formation of other reactive oxygen species and may induce oxidative stress mechanisms, including thiol-disulfide reductase and bacterioferritin. Furthermore, the perturbation of the ETC is corroborated by the expression of the ETC alternatives such as cytochrome d ubiquinol oxidase (CyoD) and formate dehydrogenase. Several membrane proteins, peptidoglycan binding proteins and Polysaccharide deacetylase upregulated upon exposure to low pH indicated the remodeling of cell wall/membrane under acid stress. Acid stress was also shown to be linked to osmotic stress as the up-regulation of the genes involved in Proline biosynthesis was observed under acid stress. The involvement of different membrane transporters to support the other mechanisms was also observed.

We also observed differential expression of several genes that coded for proteins with unknown functions and hypothetical proteins. Other bacterial RNA-seq studies have also reported the differential expression of hypothetical proteins^20,95^. The overall mechanism operated by *B*. *megaterium* G18 during acid stress based on the present study is represented in Fig. 4.

Despite the different habitat, physiology, and genetic background, the mechanisms of acid tolerance in most bacteria are similar. The responses of *B*. *megaterium* G18 cells to acid stress resemble the responses reported in other Gram-positive organisms such as *Bacillus cereus* and *B*. *subtilis*. However, several crucial differences were observed in this study in that, the *B*. *megaterium* G18 did not utilize all possible protective mechanisms, *viz*. transporting protons outwards via F1F0-ATPase, producing ammonium via urease and ADI pathway, production of biofilm, chaperon mediated protection of macromolecules. The major mechanisms adopted by *B*. *megaterium* G18 to survive during acid stress condition include the maintenance of cell integrity, alternative energy generation, GAD dependent pH homeostasis etc. Most importantly, this study indicates the involvement of proline in acid tolerance response which has not been previously reported.

## Methods

### Bacterial strain and growth condition

The *B*. *megaterium* G18^14^ was used for transcriptome analysis. Nutrient Broth (NB) and Nutrient Agar (NA) (Merck, Germany) was used to grow *B*. *megaterium* G18.

### Acid shift growth curves and acid tolerance test

Acid shift growth curves and extreme acid resistance of *B*. *megaterium* G18 was tested by growing in a moderate acid. *B*. *megaterium* G18 was cultured overnight in NB at pH 7.0. The overnight culture was diluted 100-fold in NB at pH 6.0 (adjusted with HCl) and pH 7.0. The cultures were incubated with aeration (200 rpm) at 37 °C until an optical density at 600 nm (OD_600_) of 0.3, 0.8 and 1.0. The cultures were diluted 50 fold in NB at pH 4.5 (adjusted with HCl), cultured for 4 h and optical density was measured. For each condition, three biological replicates were tested. For acid tolerance test, serial dilutions of the 4 h grown cultures were plated on NA and compared to cells plated in dilutions of the original culture at pH 7.0. Three plates from three independent cultures were scored for each condition. Change in pH of the medium during the growth of *B*. *megaterium* G18 was also recorded at an interval of 1 h till 8 h and after overnight incubation.

### RNA isolation, library preparation, and RNA sequencing

For RNA isolation, *B*. *megaterium* G18 was inoculated in NB medium at pH 7 and incubated overnight at 37 °C for 16 h. The overnight culture was diluted 100-fold in NB at pH 6.0 and cultured until an OD_600_ of 1.0. The cultures were further diluted 50 fold in NB, adjusted to pH 4.5 and incubated for 4 h at 37 °C. Five biological replicates were cultured for both non-stress (pH 7.0) and acid stress (pH 4.5) conditions. The replicate cultures were pooled, and harvested by centrifuging at 8000 rpm for 10 min and used for RNA isolation. Total RNA of *B*. *megaterium* G18 was isolated using TRI Reagent® (Sigma-Aldrich, MO, USA). The quality of the isolated RNA was confirmed by the presence of 23S and 16S bands on 1% denaturing agarose gel. Further, total RNA was quantified using Qubitfluorometer (Invitrogen™, CA, USA). The bacterial mRNA was enriched from total RNA by removing the 16S and 23S ribosomal RNAs using MICROBExpress kit (Life Technologies, CA, USA). The mRNA was then reverse transcribed into first-strand cDNA using GoScript™ Reverse Transcription kit (Promega, Madison, USA) and random hexamer primer as per manufacturer’s protocol. usingSuperScript™ Double-Stranded cDNA Synthesis Kit (Invitrogen™, CA, USA). The paired-end cDNA sequencing libraries were prepared using TruSeq Stranded mRNA Library Preparation Kit (Illumina, California, USA) as per the described protocol. Briefly, the single-stranded cDNA was converted into double-stranded cDNA and subjected to Covaris shearing followed by end-repair of overhangs resulting from shearing. The end-repaired fragments were A-tailed, adapter-ligated and then enriched by a limited number of PCR cycles. Library quantification and qualification were performed using DNA High Sensitivity Assay Kit (Invitrogen™, CA, USA). The libraries prepared from both pH 7.0 and pH 4.5 grown samples were sequenced using 2 × 150 PE chemistry on Illumina NextSeq-500 platform.

### Mapping, assembly, and differential gene expression

The raw data were initially processed to obtain clean reads by removing the adapter sequences and low-quality bases. NGS QC toolkit (v 0.30)^96^ with quality value Q > 20 was used for quality control of raw reads. The clean reads were then aligned to the reference genome of *B*. *megaterium* NBRC 15308 (Genome assembly: ASM83298v1) using the alignment software Bowtie 2^97^. Cufflinks^98^ was used to assemble the mapped reads. The fragment per kilobase of transcript per million mapped fragments (FPKM) was used to measure the expression levels for different genes and transcripts. Cuffdiff was used to analyze coverage, distribution, and differential gene expression based on FPKM normalization. Genes with false discovery rate (FDR) value <0.01 were considered as differentially expressed. Finally, CummeRbund (http://compbio.mit.edu/cummeRbund/)was used to visualize and integrate the results of the Cuffdiff analysis. Further cluster analysis of DEGs was performed using Euclidean distance method in R^99^.

### Functional annotation and biological pathway analysis

Blast2GOPRO was used to annotate the DEGs and to assign gene ontology (GO) terms. KEGG Automatic Annotation Server^100^ along with Blast2GOPRO was used to predict pathway(s) of differentially expressed genes. The BBH (Bi-directional best hit) option of KAAS annotation server was used to assign KO terms. Network analysis of significant DEGs and GO-terms was carried out in Cytoscape^101^.

### Quantitative real-time PCR (qRT-PCR) validation

A total of 31 genes belonging to different operons were chosen for confirmation of RNA-seq data by qRT-PCR. Primers used in this study along with their target genes and log_2_ fold change values are listed in Supplementary Table S1. Total RNA of *B*. *megaterium* G18 was isolated after growing the bacterium in the condition that mimics the condition of RNAseq library preparation described above using TRI Reagent® (Sigma-Aldrich, MO, USA) followed by DNase I treatment and purified using silica column (Ambion, Life technology, USA). First strand cDNA was prepared from the isolated RNA using GoScript™ Reverse Transcription kit (Promega, Madison, USA). Quantitative real-time PCR was performed on the first strand cDNA using GoTaqqPCR Master Mix (Promega, Madison, USA) in a total reaction volume of 20 μl containing 10 nM primers and 50 ng cDNA template according to the manufacturer’s instructions. Real-time PCR was performed using three biological replicates on the StepOne Plus Real-Time PCR System (Applied Biosystems, USA) with 16S rRNA gene as the reference gene^18^. The relative gene expression data were analyzed using the 2^−ΔΔCt^ method^102^.

### Qualitative analysis of urease and arginine deiminase activity

The change in pH of the medium during the growth of the isolate G18 was monitored which revealed an increase in the pH of the media (pH 4.5 to pH 6.68). Therefore, we hypothesized that the isolate may either be producing alkali to neutralize acid stress or the rise in pH may be due to the liberation of ammonia from peptone in the media. Bacteria are known to produce alkali *via* urease and arginine deiminase pathway and therefore we qualitatively analyzed urease and arginine deiminase (ADI) after growing the isolate at pH 4.5. To test the urease activity, *B*. *megaterium* G18 was grown in minimal media (Himedia, India) of pH 7.0 and pH 4.5 at 30 °C until OD_600_ of 0.8. One hundred microliters of bacterial cultures were used to inoculate 20 ml urease test broth (Himedia, India) and incubated at 30 °C without shaking. The color change in urease test broth was monitored at 24 h. The un-inoculated urease broth was taken as the negative control and *E. coli* was taken as positive control^103^. ADI activity of whole-cell lysates of *B*. *megaterium* G18 was tested based on the production of L-citrulline from L-arginine using the chemical colorimetric method^104,105^. The uninoculated reagents were used as negative controls and citrulline as the positive control. The development of an orange color was monitored and considered as the positive test.

### Biofilm production test

Formation of biofilm has been reported as a strategy of bacteria to cope with acid stress^5^. *B*. *megaterium* G18 was tested for biofilm production under acid stress using crystal violet method as described earlier^106^.

### Confocal Microscopy

In order to ascertain whether acid stress induces sporulation in *B*. *megaterium* G18, microscopic analyses were performed. Bacterial cells were grown in NB till OD_600_ of 1.0 at 37 °C and shifted to NB of pH 4.5 and pH 7.0 followed by incubating at 37 °C for 4 h with shaking. One milliliter of the cells from each culture was centrifuged and washed twice in 1 ml of 0.22 µm filtered PBS then stained using the Live/Dead® BacLight^TM^ bacterial viability kit (Molecular Probes, USA**)** following the manufacturer’s protocol. Briefly, the bacterial cells were incubated in the dark at room temperature for 15 min with 0.5 µL each of 1.67 mM Propidium iodide (PI) (red dye) and 1.67 mM SYTO 9 (green dye). *B*. *megaterium* spores were also stained similarly and used as the control. The microscopic observations were performed using a Leica TCS SPE confocal laser scanning microscope system supported by the LAS X software (Leica Microsystems, Germany).

### Intracellular proline assay

The intracellular concentration of proline in the bacterial cells was measured by the colorimetric assay using the modified method of Bates *et al*. (1973)^90,107^. Briefly, *B*. *megaterium* G18 was grown similarly as described for RNAseq library preparation and 20 mL of the bacterial culture was harvested and washed twice with phosphate buffer (pH 7.0). Cell pellet was mixed with 1 ml of 3% 5-sulfosalicylic acid and kept at room temperature overnight. The mixture was centrifuged to remove the cell debris. One milliliter of supernatant was mixed with 0.5 ml acid ninhydrin solution and 0.5 ml glacial acetic acid. The reaction mixture was incubated in boiling water (100 °C) for 1 h, and then immediately cooled on ice bath. Two milliliter of toluene was added to the reaction mixture and mixed vigorously followed by incubation at room temperature for 15 min. The upper layer was collected and the absorbance at 520 nm was measured against toluene as a blank. Standard proline at 10–300 μM range was used to prepare a standard curve.

### Statistical analysis and data submission

At least three independent replicates of each experiment were performed if not mentioned otherwise. The results of Real-time RT-PCR, intracellular proline measurements and Biofilm production tests were analyzed using student *t*-test and *p* ≤ 0.01 was considered as significant. The transcriptome sequence data were submitted to NCBI SRA (Bio-project Id: PRJNA421078).

## Electronic supplementary material

Supplementary file
