# Supplementary material for: Bacillus megaterium adapts to acid stress condition through a network of genes: Insight from a genome-wide transcriptome analysis
Source: Sci Rep. 2018 Oct 31;8:16105. doi: 10.1038/s41598-018-34221-0 (PMC6208408; doi:10.1038/s41598-018-34221-0)
Supplement: Supplementary file 1 — Supplementary file [file 41598_2018_34221_MOESM1_ESM.pdf]

Bacillus megaterium adapts to acid stress condition through a network of genes: Insight from a genome-wide transcriptome analysis

Gunajit Goswami[1,2], Debashis Panda[3], Ramkrishna Samanta[2], Robin Chandra Boro[1], Mahendra Kumar Modi[1,3], Kamal Malla Bujarbaruah[1], Madhumita Barooah[1]*

[1]Department of Agricultural Biotechnology, Assam Agricultural University, Jorhat-785013, India

[2]Department of Life-Sciences, Dibrugarh University, Dibrugarh-786004, Assam, India

[3]Distributed Information Centre, Department of Agricultural Biotechnology, Assam Agricultural University, Jorhat-785013, India

***Corresponding author's Email:** m17barooah@yahoo.co.in

Supplementary Table S1: Primers used in qRT-PCR for validation of RNAseq results along with the target gene and log2 fold change values

Sl. No.	Primer Name	Seuences(5´-3´)	Tm (°C)	Target Gene	Log2 fold change in RNAseq analysis
1	Gad F	TAAGCAGGAACTTGCCATCC	65.0	BG04_4844 (encode Glutamate decarboxylase)	2.19522*
	Gad R	CTACGTGGATGGAGCCTGAT	66.0		
2	proC-F	GGAAGTGGACCCGCTTATTT	63.0	*proC* (encodes pyrroline-5-carboxylate reductase)	2.93785*
	proC-R	TCGGCGTTTCATCTCTTTCC	64.0		
3	BG04_015-F	AAACAAGCACATTTGCCTAAG	62.5	BG04_015; Encodes hypothetical protein)	Exclusively upregulated at pH 4.5*
	BG04_015-R	TTTCTTCATTTCGGCGATAGTA	62.8		
4	proA-F	ACTGGGAACTGCCACATTTA	63.0	*proA*; encodes glutamate-5-semialdehyde dehydrogenase	ND
	proA-R	CTCGTAGCGCTTGAACAATAATC	62.0		
5	proB-F	GGCGTGCCAAACATCTTAAC	64.0	*proB*; encodes gamma-glutamyl kinase	ND
	proB-R	GCGGCATCTTCGACTACTATT	63.0		
6	BG04_4711-F	GCTTTCCGTTGATGGCTTTA	62.5	BG04_4711; encodes hypothetical protein	5.22182*
	BG04_4711-R	GATCTATAGAGGCCGGTTGG	64.0		
7	BG04_4989-F	CCAGCTGAATAGAATCAAACAC	64.0	BG04_4989; encodes hypothetical protein	2.90606*
	BG04_4989-R	GCTGAACTAACCTGCTTAAATG	65.0		
8	BG04_4673-F	CATATGTTAGAGGAAGCACAAAG	58.4	BG04_4673; encodes hypothetical protein	4.67923*
	BG04_4673-R	TCAGAGTGACTTAGCATTAGAT	60.6		
9	BG04_2742-F	GTCACTGCTGGGTATGAAG	61.0	BG04_2742; encodes hypothetical protein	-3.91892*
	BG04_2742-R	CAGTGAAGTAACAATCCCTAGT	62.0		
10	BG04_3376-F	GCATCTGCACTGGGAATTA	62.0	BG04_3376; encodes hypothetical	-3.07018*

	BG04_3376-R	CTGAACCATCTAGACCATCAAC	62.0	protein	
11	BG04_4142-F	TGCGAAATTGGAGGAAGTAG	62.0	BG04_4142;encodes hypothetical protein	4.2937*
	BG04_4142-R	GGCAACTGACTTATGACTATGA	61.0		
12	cyoD-F	TCCTCTGCTGCTGGTATT	62.0	*cyoD*; encodes cytochrome o ubiquinol oxidase subunit IV	Exclusively upregulated at pH 4.5*
	cyoD-R	TCCGAGTATTAAAGCCATCAC	61.0		
13	cysW-F	CCACTCATGCAAGCTCAA	62.0	*cysW*; encodes sulfate ABC transporter permease subunit	3.02418*
	cysW-R	CCAGACACAACGGAAACA	62.0		
14	UreC-F	TCCGAATACGTAGGATCAGTAGAA	63.0	*ureC*; encoded urease subunit alpha	ND
	UreC-R	CGCGTTAGGATCTCCCATTAAG	62.0		
15	Hsp70F	TCGACCTGGGTACTACCAACT	61.0	*dnaK*; encodes Molecular chaperone	ND
	Hsp70R	CAGAGTGTTTTGCGGGTTCG	62.0		
16	BG04_1586-F	GGATTAAACACCCACCCAAAC	52.0	BG04_1586; encodes cys/Met metabolism PLP-dependent enzyme family protein (involved in cysteine biosynthesis)	-0.0518726 (ns)
	BG04_1586-R	GGCTTTCAATGGCTCCTAAAC	52.0		
17	BG04_1587-F	TTGAGCCTACTGCTGGAAATAC	53.0	*mccA*; encodes cystathionine beta-synthase (involved in cysteine biosynthesis)	0.434873 (ns)
	BG04_1587-R	CCAGCTTCAGTAGGTGTTTGA	52.0		
18	BG04_1589-F	TGTAGCAGACCGTTCAATAGG	55.0	*yrrT*, encodes putative AdoMet-dependent methyltransferase (involved in cysteine biosynthesis)	-0.354128 (ns)
	BG04_1589-R	AAAGTCTCCTGGTGTGACTAA	55.0		
19	BG04_3926-F	TGGGACGCTAGTACTCCTATT	52.0	BG04_3926; encodes HAD hydrolase(involved in cysteine biosynthesis)	0.774583 (ns)
	BG04_3926-R	GGGATATAAAGCACCTTTCCC	52.0		

20	BG04_4883-F	TAGCAGGTCTTGTGGTTGTAAA	53.0	BG04_4883; encodes cys/Met metabolism PLP-dependent enzyme family protein (involved in cysteine biosynthesis)	0.399613 (ns)
	BG04_4883-R	CCTCTGTCTCTTCCATTCTGAT	53.0		
21	BG04_1588-F	GCGGTGTGGAGGTTGTATTA	52.0	*mtnN*; encodes adenosylhomocysteine nucleosidase (involved in cysteine biosynthesis)	ND
	BG04_1588-R	CCGCATCTCCTACGTTTAAC	52.0		
22	BG04_1852-F	GGTGTAGACCAAGCGTTAGAAG	55.0	*metK*; encodes methionine adenosyltransferase (involved in cysteine biosynthesis)	ND
	BG04_1852-R	TAGACGGCGAGCAAGTTTATG	55.0		
23	BG04_1863-F	GCGACGGCGTTGTAAATAAAT	53.0	*luxS*; encodes S-ribosylhomocysteine lyase (involved in cysteine biosynthesis)	ND
	BG04_1863-R	TTGACATCCCATAGGCGAAAT	52.0		
24	BG04_1830-F	GCGAGACACCTCTTGTTAAG	55.0	*cysK*; encodes cysteine synthase A (involved in cysteine biosynthesis)	ND
	BG04_1830-R	TCCGCTCGTTGGTTCAATA	55.0		
25	BG04_2388-F	CACGGGATGGGAATTGTTATTG	53.0	*cysE*; encodes serine O-acetyltransferase (involved in cysteine biosynthesis)	ND
	BG04_2388-R	CGTAATCGACCCTAGCACTTTA	53.0		
26	hpr-F	CGGCAAATTCCCTGAGTTTATG	53.0	*hpr*, encodes transcriptional regulators Hpr	Exclusively upregulated at pH 4.5*
	hpr-R	GTCTCGTGGTGGTTCTCTTTAT	53.0		
27	RRf2-F	CTAGGCGGCGGTTATACTTTAG	53.0	BG04_20; encodes Rrf2 family transcriptional regulator	Exclusively upregulated at pH 4.5*
	RRf2-R	TCGTCCATCACTGTCTGAATG	55.0		
28	Spo0E-F	TGAATCGAATTGAAGAATGCAG	52.0	BG04_2465 encodes Spo0A-P phosphatase	4.52514*
	Spo0E-R	CCATCTAGCTCTGAACTGACTC	53.0		

29	spo0A-F	TCATCTTCTTCCATGCGTTC	50.0	*spo0A*, encodes transcriptional regulator	ND
	spo0A-R	ACCCAAGCAGCTCAATATCC	52.0		
30	BG04_2120-F	GGAGCTTGAGCGAAATACA	60.0	BG04_2120, encodes flotilin	-3.09691
	BG04_2120-R	CGTTCAATGATGCGGATTTG	61.0		
31	16S-F	CTCTAAGGAGACTGCCGGTG	64.0	*16S rRNA*, encodes16S ribosomal RNA	NA
	16S-R	CGGACTACGATCGGTTTTGT	64.0		

*- indicates significant; ns- non-significant; ND- not detected; NA- not applicable

Supplementary Table S2: Data were generated by sequencing of the two *B. megaterium* cDNA samples, namely, pH 7.0 and pH 4.5 with Illumina Nextseq 500 and their mapping rates with the reference genome.

**Sample Name**	**No. of Raw reads**	**No. of Quality reads**	**Mapped reads (%)**
pH-7.0	13859399	10504233	73.8%
pH-4.5	12443960	10505911	66.42%

Supplementary Table S3: Differential Expression pattern of genes, their Blast2GO description and test statistics

Gene_Id	Description(Blast2GO)	FPKM_ pH-7.0	FPKM_ pH-4.5	Log_2Fold _Change	p-value	q-value	e-Value	sim(%)
BG04_15	family transcriptional regulator	0	32.5881	1.7977E+308	5.37247E-05	0.0040112	3.3E-71	94.10%
BG04_643	MULTISPECIES: hypothetical protein	0	77.2071	1.7977E+308	0.0016035	0.0445193	1.1E-25	97.80%
cyoD	cytochrome o ubiquinol oxidase subunit iv	0	32.9519	1.7977E+308	8.73959E-05	0.00561468	4.3E-43	98.30%
BG04_5405	hypothetical protein BMWSH_2308	0	2360.54	1.7977E+308	5.37247E-05	0.0040112	2.9E-23	85%
BG04_20	rrf2 family transcriptional regulator	0	7.45082	1.7977E+308	0.000424381	0.0160596	1.6E-99	97.50%
BG04_149	uncharacterised protein	0	8762.85	1.7977E+308	6.55096E-05	0.00458153	2E-18	87.17%
BG04_1585	yrhc-like family protein	0	66.4556	1.7977E+308	0.000140332	0.0074976	3.1E-37	92.90%
hpr	transcriptional regulator hpr	0	21.7286	1.7977E+308	4.00476E-05	0.00330243	2.5E-86	94%
BG04_3928	yesk-like family protein	0	28.8314	1.7977E+308	0.000198014	0.00943127	1.2E-36	91.30%
BG04_4817	hypothetical protein	0	13.3035	1.7977E+308	2.99585E-05	0.00265247	4.5E-100	98.10%
BG04_5856	hypothetical protein	0	11.5374	1.7977E+308	0.000483519	0.017347	2.5E-76	74.56%
BG04_4711	MULTISPECIES: hypothetical protein	1.75437	65.4708	5.22182	3.49365E-10	2.75749E-07	5.3E-109	98.90%
BG04_4673	hypothetical protein	104.534	2678.22	4.67923	5.9952E-12	3.31235E-08	2E-55	93.20%
BG04_2465	spo0a-p phosphatase	93.6132	2155.46	4.52514	1.55094E-10	1.42815E-07	2.8E-29	73.90%
BG04_4142	cupin	1.18685	23.2771	4.2937	4.91511E-11	6.789E-08	0	91.30%
BG04_125	hypothetical protein BMQ_3197	304.512	4199.09	3.78551	1.97693E-11	3.64085E-08	3.2E-32	100%
BG04_4670	protein containing a divergent version of the methyl-accepting chemotaxis-like domain protein	64.0404	749.32	3.54853	1.23048E-09	7.55379E-07	1.3E-42	99.60%
BG04_4536	hypothetical protein	4.61942	53.063	3.52192	4.29577E-07	0.000107882	9.3E-83	87.60%
BG04_3885	hypothetical protein DJ91_1387	46.339	526.067	3.50495	0.00170759	0.046475	2.5E-20	94.25%
ydaG	general stress protein	279.501	3031.13	3.43893	8.33137E-05	0.00541539	3.1E-101	91.80%
BG04_4570	holin	1.48738	15.6976	3.39969	3.02453E-05	0.00265247	2.6E-99	85%
BG04_3608	MULTISPECIES: hypothetical protein	21.572	214.546	3.31406	5.49661E-05	0.00404917	1.2E-39	82.60%
BG04_3847	bacterioferritin	40.4365	392.604	3.27935	2.5133E-07	7.71444E-05	1.7E-96	94.40%
BG04_4542	hypothetical protein	1.42922	12.7391	3.15597	0.000113019	0.00657293	8.3E-115	100%

BG04_5651	penicillin-binding protein	30.3366	263.901	3.12087	0.000141131	0.0074976	0	97%
BG04_75	hypothetical protein	155.577	1322.61	3.08768	1.00783E-05	0.00113638	5E-70	96.60%
cysW	sulfate abc transporter permease subunit	0.682064	5.54872	3.02418	0.000219995	0.00999727	2.3E-173	98.80%
BG04_2806	---NA---	423.063	3373.5	2.9953	1.73268E-06	0.000308808	-	-
BG04_562	hypothetical protein	9.14863	70.3487	2.9429	2.40644E-05	0.00221593	1.7E-60	92.30%
BG04_5391	hypothetical protein	2.45583	18.8686	2.94171	2.29552E-05	0.00214962	4.8E-105	94%
proC	pyrroline-5-carboxylate reductase	1.90031	14.5614	2.93785	6.16515E-07	0.00013625	0	99.40%
BG04_220	general stress protein	1.67461	12.6437	2.91653	0.000385792	0.0148888	8.1E-47	97.80%
BG04_4989	hypothetical protein	17.48	131.025	2.90606	9.74343E-08	3.16662E-05	1.9E-86	97.70%
BG04_4569	hypothetical protein	1.2255	9.03158	2.88161	0.000452982	0.0167578	1.7E-136	66.90%
BG04_951	acyltransferase	5.38728	39.6646	2.88022	0.000000313	9.10272E-05	0	91.90%
BG04_4915	formate dehydrogenase	35.508	257.667	2.85929	4.32746E-06	0.000646195	1.4E-112	98.20%
acyP	acylphosphatase	97.4571	693.942	2.83198	1.51723E-06	0.00028362	3.8E-62	99.40%
BG04_573	membrane protein	11.8225	77.18	2.70669	1.57364E-05	0.00161484	1.1E-23	95.30%
BG04_4951	MULTISPECIES: hypothetical protein	5.31487	34.5618	2.70107	0.000110926	0.00651988	2.2E-76	98.70%
BG04_3867	membrane protein	0.863428	5.50389	2.6723	0.00120443	0.0367908	2.4E-126	99.40%
BG04_53	hypothetical protein	49.199	298.644	2.60173	9.27932E-06	0.00108259	6E-86	98.60%
BG04_3661	nad-dependent epimerase dehydratase	5.73277	34.7965	2.60164	0.00181082	0.0488038	1.4E-46	93.80%
BG04_4721	membrane protein	2.27741	13.598	2.57793	0.000223237	0.00999727	1.3E-101	98.80%
BG04_4566	phage minor structural protein	2.23883	13.1891	2.55853	2.2012E-06	0.000368533	0	65.50%
BG04_3189	hypothetical protein DJ91_2084	391.835	2307.36	2.55792	0.00137291	0.0405496	5.9E-58	99.20%
BG04_4845	hypothetical protein	12.5154	72.4591	2.53346	2.62229E-06	0.000426121	1.6E-124	98.80%
BG04_4538	transcriptional regulator	2.79643	15.898	2.50719	0.000341875	0.0138887	2.7E-97	92.50%
BG04_778	MULTISPECIES: hypothetical protein	688.125	3904.57	2.50442	0.00142534	0.0408032	1.4E-56	98.75%
BG04_3459	uncharacterised protein	9.67077	54.7358	2.50078	1.70298E-05	0.00171072	7.5E-74	92.80%
BG04_5052	hypothetical protein	1.99846	11.2659	2.495	6.80755E-05	0.00463618	1.9E-160	98.30%
BG04_1220	methylthioribose kinase	7.87784	43.8836	2.47781	8.73822E-06	0.00106941	6.3E-90	95.20%
BG04_3658	sporulation family protein	6.41732	35.2932	2.45935	4.92572E-06	0.000709496	2.7E-123	94.40%
BG04_4568	carbohydrate-binding domain protein	2.65922	14.3768	2.43467	2.19731E-05	0.00212985	0	75.40%
BG04_4556	phage head-tail adapter protein	2.91334	15.6861	2.42874	0.0166564	0.193333	1.1E-86	83.20%
BG04_2964	dna methyltransferase	71.4731	380.07	2.41079	1.90621E-05	0.00188068	1.8E-66	87.40%
BG04_1947	phosphohydrolase	8.41085	44.5871	2.4063	6.91747E-06	0.000932171	6.3E-128	96.90%
BG04_4693	MULTISPECIES: hypothetical	94.1674	494.884	2.39379	1.28031E-05	0.001387	2.4E-44	88.20%

	protein							
BG04_5557	chy zinc finger	5.42975	28.3292	2.38333	0.000727504	0.0246455	8.1E-78	98.20%
BG04_2814	l-lactate dehydrogenase	18.2477	95.1486	2.38247	0.000223968	0.00999727	0	99.50%
BG04_4030	hypothetical protein	6.50527	33.8227	2.37831	8.42746E-06	0.00106941	6E-167	99%
BG04_4567	minor structural protein 1	3.34157	17.3639	2.37749	8.90365E-06	0.00106941	0	76.50%
BG04_4956	gnat family n-acetyltransferase	4.25096	21.9601	2.36902	0.000162466	0.00819899	1.2E-99	99.20%
isp	serine protease	19.8289	100.427	2.34047	0.000372331	0.0146938	0	99.30%
BG04_3445	capsular biosynthesis protein	4.83755	24.137	2.3189	1.42243E-05	0.00151133	2E-172	98.90%
BG04_4813	thiol-disulfide oxidoreductase dcc	5.7057	28.3452	2.31263	6.98067E-05	0.00464677	3.6E-98	91.20%
BG04_4563	phage tail tape measure protein	3.06181	15.0922	2.30134	0.000196748	0.00943127	0	59.30%
BG04_5281	cysteine desulfurase	11.3566	54.745	2.2692	0.000222939	0.00999727	0	93.50%
BG04_5540	peptidase	2.55754	12.3127	2.26731	2.29345E-05	0.00214962	0	97.90%
BG04_4972	pd-(d e)xk nuclease superfamily protein	1.68161	8.01535	2.25292	0.000106826	0.00648585	0	98.70%
BG04_5029	hypothetical protein	0.97747	4.65379	2.25128	0.00136996	0.0405496	0	96.50%
yhaH	membrane protein	11.6551	54.7608	2.23218	4.23705E-05	0.0034426	4.4E-78	98.90%
BG04_5105	peptidase	3.77137	17.4696	2.21169	6.88084E-05	0.00463618	1.3E-139	98.40%
BG04_4844	glutamate decarboxylase	0.928972	4.25432	2.19522	0.000159781	0.00818337	0	99.10%
BG04_4483	molybdenum abc transporter atp-binding protein	2.31652	10.5573	2.18821	8.24213E-05	0.00541539	0	99.30%
BG04_1000	hypothetical protein	9.85215	44.443	2.17345	3.81721E-05	0.00319547	8.3E-91	98.30%
BG04_4148	membrane protein	18.2973	82.0735	2.16529	9.61559E-05	0.00601274	1.6E-13	97.80%
BG04_2888	heme-degrading monooxygenase	25.5828	113.563	2.15025	0.000135244	0.00739824	6.3E-122	99.30%
yokD	aac family n-acetyltransferase	6.52897	28.4677	2.1244	5.79068E-05	0.00420967	1.7E-179	98.50%
BG04_5568	methyltransferase	2.41877	10.2468	2.08283	0.00017518	0.0086417	0	98.60%
yocH	peptidoglycan-binding protein	12.1129	50.2806	2.05346	0.00038382	0.0148888	5.4E-155	98.10%
BG04_21	lipo [Bacillus megaterium NBRC 15308 = ATCC 14581]	7.29388	29.8116	2.03112	0.000110492	0.00651988	2.1E-151	98.90%
BG04_4053	stress protein	6.14031	25.0406	2.02789	0.000195186	0.00943127	1.2E-88	99.40%
BG04_5451	alpha beta hydrolase	8.56579	34.8001	2.02243	0.000177063	0.00865726	0	98.80%
BG04_4289	polysaccharide deacetylase	20.2362	82.1834	2.02191	0.00118826	0.0367908	0	98.50%
BG04_204	transporter	1.2584	5.10402	2.02005	0.00138888	0.0405496	0	98.80%
BG04_4671	alpha beta hydrolase	4.32982	17.5515	2.01922	0.000122924	0.00693014	0	97.90%
BG04_5355	membrane protein	32.3359	130.897	2.01723	0.00030004	0.012951	1.3E-12	99.67%
BG04_2965	crp fnr family transcriptional regulator	5.80916	23.2737	2.0023	0.000308402	0.0132087	2.6E-106	99.40%
BG04_4411	gcn5 family acetyltransferase	7.1461	28.6109	2.00133	0.000163238	0.00819899	8.6E-128	98.60%

BG04_5049	cxxc_20_cxxc family protein	152.166	605.491	1.99246	0.00109054	0.0342342	3.8E-67	81%
BG04_4991	isochorismatase	4.82113	19.1373	1.98894	0.000218124	0.00999727	1.6E-146	98.70%
BG04_5276	membrane protein	7.18264	28.3941	1.983	0.000159921	0.00818337	1E-123	95.20%
BG04_659	mfs transporter	1.15883	4.56208	1.97702	0.000692665	0.02377	0	98.40%
BG04_264	multidrug transporter	1.30913	5.13374	1.9714	0.00184007	0.0493514	0	98%
BG04_10	l-asparaginase 1	2.30808	9.01328	1.96536	0.000261612	0.0114715	0	99.10%
BG04_1147	asp23 family protein	7.55572	29.3391	1.95718	0.000838519	0.0279085	5.4E-80	98.70%
BG04_3795	hypothetical protein	15.7384	60.1246	1.93366	0.000228803	0.0101131	8.6E-88	98.30%
BG04_818	tetratricopeptide repeat family protein	6.33624	24.0397	1.92372	0.000338354	0.0138475	6.4E-119	98.90%
BG04_755	methyltransferase	9.01274	34.1574	1.92216	0.000266016	0.0115727	0	97.60%
corA	magnesium and cobalt transport protein	2.3737	8.94637	1.91417	0.000370163	0.0146938	0	98.50%
BG04_4532	phage rha family protein	3.35947	12.5753	1.90429	0.000463747	0.0169234	2.7E-166	72.20%
BG04_4752	gamma-aminobutyrate permease	3.65971	13.6854	1.90284	0.00031153	0.01324	0	99.80%
BG04_5344	diguanylate cyclase	2.963	11.0221	1.89527	0.000329123	0.0136722	0	97.60%
BG04_137	membrane protein	6.07851	22.4275	1.88348	0.000332473	0.0137083	6.2E-144	98.30%
BG04_607	nadph-dependent fmn reductase	8.31434	29.6615	1.83492	0.000438966	0.0163871	5E-125	99.60%
BG04_5342	capsular biosynthesis protein	7.44924	26.068	1.80711	0.000903108	0.029351	0	98.30%
nprM	bacillolysin	5.53678	19.3698	1.80669	0.00132466	0.0397759	0	99.10%
BG04_3482	iron-sulfur cluster biosynthesis family protein	14.5205	49.6236	1.77293	0.00113501	0.035429	6.1E-71	98.70%
BG04_4921	hypothetical protein	1.67805	5.53353	1.72141	0.00196887	0.052048	0	85%
BG04_4843	hypothetical protein	3.8857	12.4497	1.67986	0.00120527	0.0367908	4.4E-95	99.90%
BG04_3436	tyrosine protein phosphatase	4.03129	12.7516	1.66137	0.00157133	0.0440689	0	94.40%
BG04_2930	beta-ketoacyl-acp reductase	8.05969	25.4568	1.65926	0.00135294	0.0404053	1.5E-180	98.50%
BG04_307	3-oxoadipate enol-lactonase	3.90988	12.1363	1.63414	0.00168161	0.0459947	0	98.70%
BG04_5214	hypothetical protein	2.35568	7.28127	1.62804	0.00164292	0.0451598	0	94.30%
BG04_4533	replication protein	5.35483	16.1826	1.59554	0.00202097	0.0531585	0	70.80%
BG04_5995	hypothetical protein	37.2804	12.0462	-1.62984	0.00192312	0.0510829	8E-104	86.80%
BG04_2371	4-amino-4-deoxychorismate lyase	20.2153	6.44091	-1.65011	0.00149853	0.0424584	0	98.10%
BG04_2114	chemotaxis protein	25.7467	8.14498	-1.66041	0.00140197	0.0405543	8E-165	95.10%
BG04_371	d-alanyl-d-alanine carboxypeptidase	19.1558	5.89674	-1.69979	0.00141285	0.0406563	1.9E-139	99%
BG04_1042	3-ketoacyl-acp reductase	10.9219	3.34372	-1.7077	0.00160043	0.0445193	1.8E-153	99.70%
hprK	serine kinase	22.1738	6.71455	-1.72349	0.0010241	0.0323324	0	97.60%

BG04_1969	had family hydrolase	30.0688	9.05614	-1.7313	0.00100533	0.0321067	3.2E-178	92%
BG04_5991	MULTISPECIES: hypothetical protein	105.793	31.704	-1.7385	0.00101134	0.0321129	8.9E-69	73.40%
flgF	flagellar basal body rod protein	43.6278	13.0606	-1.74003	0.0016175	0.0446834	0	92.80%
BG04_6012	mobilization protein	126.415	37.4661	-1.75451	0.000921806	0.0297835	1.6E-79	96.90%
fabD	malonyl -acyl carrier protein transacylase	41.969	12.4061	-1.75827	0.00203013	0.0531585	0	99.20%
rsbT	serine threonine protein kinase	50.9036	14.9841	-1.76433	0.00087768	0.0286934	1.5E-90	93.20%
BG04_5005	spore protein n	2573.54	748.449	-1.78178	0.00120064	0.0367908	2.2E-25	91.70%
BG04_981	transposase	13.5122	3.92157	-1.78476	0.000631187	0.0218698	0	96%
fmnP	riboflavin transporter	16.8264	4.86857	-1.78916	0.000963834	0.0309604	6E-115	98.30%
BG04_2093	flagellar assembly protein	63.7784	18.433	-1.79078	0.000875533	0.0286934	5E-74	97.80%
BG04_2158	membrane protein	17.5759	4.91198	-1.83922	0.00073156	0.0246455	3.6E-106	95.40%
BG04_2616	phosphatidic acid phosphatase	33.7931	9.40325	-1.8455	0.000434885	0.0163452	1.8E-131	98.20%
sdaAB	serine dehydratase	23.9815	6.55987	-1.87018	0.000388052	0.0148888	4.2E-145	95.70%
greA	transcription elongation factor	30.3412	8.23966	-1.88062	0.000465585	0.0169234	7.1E-95	97.50%
BG04_3278	thioredoxin	17.8806	4.80938	-1.89447	0.000568344	0.0201289	5.9E-132	96.80%
rpsN1	30s ribosomal protein s14	341.896	90.8416	-1.91213	0.00138294	0.0405496	6E-36	99.20%
fliS	flagellar protein	38.7837	10.1143	-1.93906	0.000377086	0.0147759	4.6E-94	99.50%
BG04_942	yodl-like family protein	45.5162	11.7589	-1.95263	0.000869654	0.0286934	2.9E-75	83.60%
BG04_5674	transposase	15.6916	4.04224	-1.95677	0.000205818	0.00971919	0	96%
rplN	50s ribosomal protein l14	219.957	56.2854	-1.96639	0.000577103	0.0203089	3.8E-63	98.70%
rpiB	ribose-5-phosphate isomerase	24.858	6.32755	-1.97399	0.000360705	0.0144413	8.6E-110	99.60%
BG04_2069	membrane protein	102.358	25.4331	-2.00884	0.000159965	0.00818337	3.9E-36	95.60%
BG04_5548	duf350 domain-containing protein	28.1154	6.88449	-2.02994	0.000348259	0.0140448	1.8E-80	99.90%
BG04_758	d-threo-aldose 1-dehydrogenase	15.8807	3.88154	-2.03257	0.000120028	0.00683665	0	99%
BG04_1263	1-acyl-sn-glycerol-3-phosphate acyltransferase	27.2627	6.61191	-2.04379	0.000140427	0.0074976	3.1E-133	99.60%
BG04_3377	uncharacterised protein	134.733	32.2209	-2.06403	0.000716124	0.0244234	6.4E-48	81.60%
fabH	3-oxoacyl-acp synthase	95.6074	22.8301	-2.06618	0.00174783	0.0473372	0	94.70%
rpsR	30s ribosomal protein s18	415.243	99.1036	-2.06694	0.000131786	0.00728118	1.6E-40	99.80%
rpsH	30s ribosomal protein s8	445.623	105.991	-2.07189	0.00187098	0.0499379	1.8E-90	98.80%
BG04_2884	ABC transporter-associated	132.207	31.3533	-2.07611	0.00131109	0.0397759	1.4E-140	99.30%
fabH	3-oxoacyl-acp synthase	22.5255	5.32681	-2.08022	8.97439E-05	0.00569925	0	99.30%
fliJ	flagellar export protein	45.7065	10.7808	-2.08394	0.000109775	0.00651988	9.9E-75	92.60%
BG04_2777	uncharacterised protein	118.626	27.569	-2.1053	0.00132016	0.0397759	1.8E-43	97.40%
gdhB	sugar dehydrogenase	61.6533	14.0864	-2.12987	0.000216107	0.00999727	1.4E-176	99.90%

sigW	rna polymerase sigma factor	83.8775	18.9008	-2.14984	0.000114267	0.00657631	6.7E-110	96.90%
rpsQ	30s ribosomal protein s17	295.76	65.5056	-2.17474	5.95122E-05	0.00427019	7.7E-56	98.80%
BG04_2668	zinc transporter	41.3491	8.84864	-2.22433	6.76032E-05	0.00463618	0	98.90%
rpsO	30s ribosomal protein s15	182.157	38.9636	-2.22499	4.84836E-05	0.00377284	2.2E-47	95.90%
infA	translation initiation factor if-1	164.203	34.9756	-2.23106	0.000477144	0.0172302	1.3E-45	98.80%
BG04_2599	pasta domain protein	96.9151	20.4065	-2.24769	0.000147195	0.00774525	1.4E-133	93.80%
BG04_1762	membrane protein	13.81	2.75111	-2.32763	0.00139447	0.0405496	3E-76	96.90%
rpsF	30s ribosomal protein s6	472.681	92.0497	-2.36038	4.47214E-05	0.00358095	5.2E-62	96.70%
BG04_3935	hypothetical protein	16.8299	3.22425	-2.38399	2.90529E-05	0.00263143	3E-129	97.60%
prsA1	foldase	23.5426	4.43202	-2.40924	7.70655E-06	0.00101378	0	99.10%
BG04_3967	hypothetical protein	30.2998	5.61905	-2.43091	9.68568E-05	0.00601274	3.6E-79	95.40%
BG04_1299	-like protein	126.121	23.0088	-2.45455	0.000224373	0.00999727	0	99.80%
BG04_943	phosphoesterase	34.8338	6.24484	-2.47975	5.0082E-06	0.000709496	1.6E-109	98.80%
rplJ	50s ribosomal protein l10	430.864	75.62	-2.51039	0.000397531	0.0151473	3.3E-112	94%
BG04_4380	duf4825 domain-containing protein	27.9334	4.76123	-2.55259	5.19733E-06	0.000717882	5.9E-133	97.10%
speB	agmatinase	240.809	40.7661	-2.56245	0.000788473	0.0264019	0	98.70%
rplX	50s ribosomal protein l24	664.34	111.469	-2.57528	4.98197E-05	0.00382297	5.9E-65	97.30%
rplU	50s ribosomal protein l21	265.79	42.7298	-2.63698	2.20084E-06	0.000368533	4E-65	94.60%
BG04_763	gcn5 family acetyltransferase	103.624	16.0132	-2.69402	1.33158E-06	0.000262749	2.6E-116	97.40%
BG04_3965	membrane protein	19.0407	2.9385	-2.69593	6.43233E-05	0.00455624	3.4E-87	97.50%
BG04_3158	betaine-aldehyde dehydrogenase	92.3002	13.977	-2.72328	9.92663E-05	0.00609385	0	99.20%
BG04_3964	hypothetical protein	49.4165	7.13299	-2.79241	4.17654E-07	0.000107882	2.2E-137	97.80%
rpsT	30s ribosomal protein s20	255.597	36.8784	-2.79302	7.17467E-07	0.000152462	5.8E-54	92.10%
BG04_5522	sporulation protein	349.633	49.8316	-2.81071	0.000454964	0.0167578	0	98.80%
cwlK	peptidase m15	11.043	1.50956	-2.87093	0.000611304	0.0213763	3.2E-125	97.90%
BG04_2000	conserved protein	115.734	14.7155	-2.97541	0.000495309	0.0176554	4.7E-56	98.30%
rplS	50s ribosomal protein l19	550.556	69.7739	-2.98013	2.75594E-06	0.000435044	5.6E-74	99%
cspB	cold-shock protein	311.22	39.0822	-2.99335	3.67266E-05	0.00312176	4.2E-38	98%
BG04_3159	putrescine aminotransferase	211.557	26.4945	-2.99728	0.000128773	0.00718657	0	99.20%
BG04_304	hypothetical protein	60.7816	7.38524	-3.04092	6.12781E-07	0.00013625	1.4E-87	98.40%
BG04_3376	flagellin domain-containing protein	129.34	15.3999	-3.07018	0.000046311	0.00365526	0	80.70%
BG04_2120	flotillin	356.714	41.6925	-3.09691	0.000318547	0.0134349	0	99.50%
fabG	beta-ketoacyl-acp reductase	111.987	12.8485	-3.12366	3.61642E-07	9.99035E-05	1.9E-164	94.20%
phoU	family transcriptional regulator	59.3605	6.75118	-3.13629	2.6898E-08	1.06151E-05	6.3E-152	91.30%
BG04_284	hypothetical protein	180.91	20.3555	-3.15178	1.07574E-05	0.00118869	0	96.40%

BG04_3378	motility protein	153.905	16.8584	-3.19049	0.000327982	0.0136722	9.1E-43	93.90%
rpmD	50s ribosomal protein l30	701.401	75.7652	-3.21063	0.00000154	0.00028362	1E-34	96.50%
dps2	dna starvation stationary phase protection protein	135.739	14.6276	-3.21407	1.97692E-08	8.40191E-06	7.2E-107	98.90%
rsiW	anti-sigma factor	215.334	22.4746	-3.26021	0.000000539	0.000129468	7E-138	96.90%
BG04_3727	flagellar basal body rod protein	593.037	51.6437	-3.52146	0.000000033	1.21504E-05	6.5E-75	97.80%
cspB	cold-shock protein	361.416	31.2658	-3.53101	8.67509E-06	0.00106941	7.9E-38	97.40%
acpP	acyl carrier protein	1034.97	86.1729	-3.58621	2.82672E-09	1.56176E-06	7.6E-24	95.80%
BG04_2747	ecsc family protein	110.035	9.1424	-3.58924	6.52698E-09	3.00513E-06	0	99.70%
rplL	50s ribosomal protein l7 l12	439.743	36.0726	-3.60769	6.2565E-09	3.00513E-06	1.4E-44	94%
BG04_2684	alanine acetyltransferase	28.6422	2.07815	-3.78477	8.72764E-08	3.01376E-05	4.8E-127	95.50%
rplR	50s ribosomal protein l18	2931.62	209.221	-3.8086	3.22956E-06	0.000495648	1.3E-69	93.50%
BG04_3728	modulator protein	3062.66	202.801	-3.91665	3.51708E-05	0.00303623	1E-127	98.90%
BG04_2742	membrane protein	217.819	14.4007	-3.91892	9.44729E-10	6.52453E-07	5.9E-48	96.60%
BG04_289	psb32 and molo-1 founding s of phosphatase family protein	341.581	22.1875	-3.94441	0.000015783	0.00161484	1.8E-139	98.80%
BG04_303	transcriptional regulator	89.9316	5.50773	-4.0293	1.87368E-11	3.64085E-08	2.5E-153	99.30%
BG04_3730	two-component sensor histidine kinase	1119.49	53.4524	-4.38844	9.40527E-06	0.00108259	0	99.10%
BG04_3119	protein liag	231.164	9.61588	-4.58735	6.91132E-11	7.637E-08	0	98.60%
BG04_2235	histidine kinase	294.197	11.7533	-4.64564	7.74231E-07	0.000158431	0	98.10%

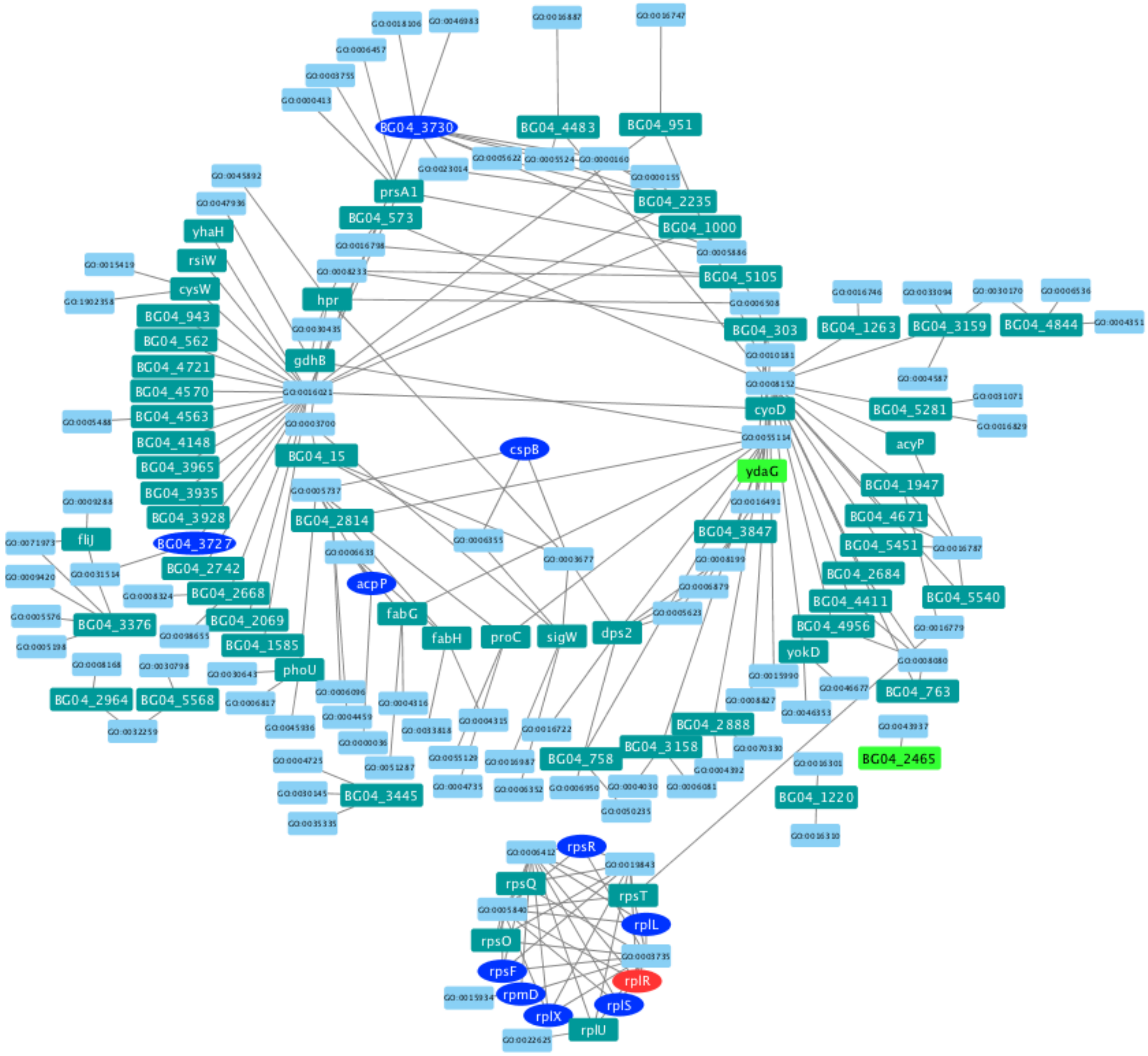

Supplementary Figure S1: Gene ontology network of significantly differentially expressed genes. (Color code: Red- Cluster 1; Blue- Cluster 2; Cyan-Cluster 3; and Green- Cluster 5

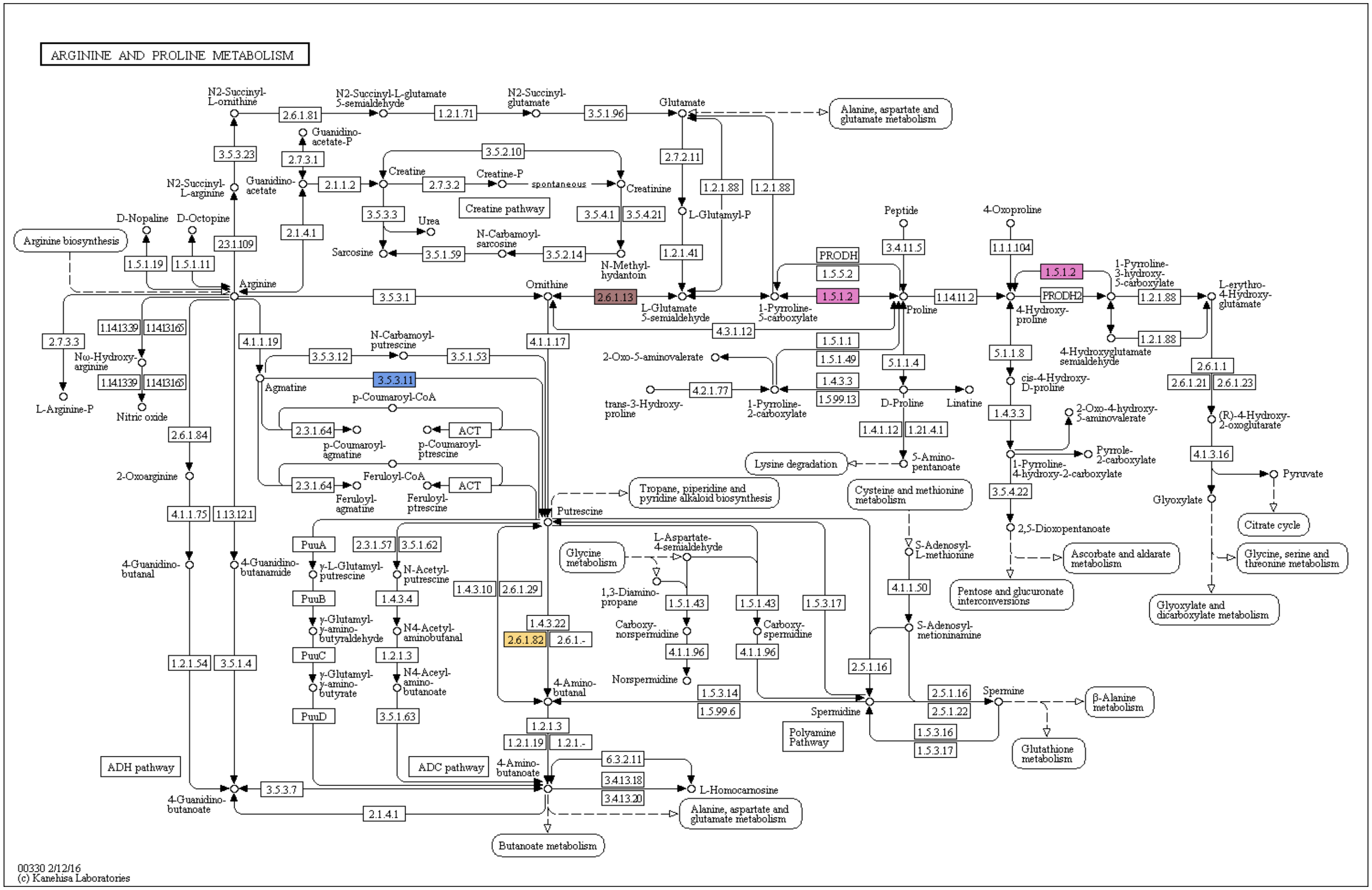

Supplementary Figure S2: KEGG map of Arginine and proline metabolism pathway[1–4]

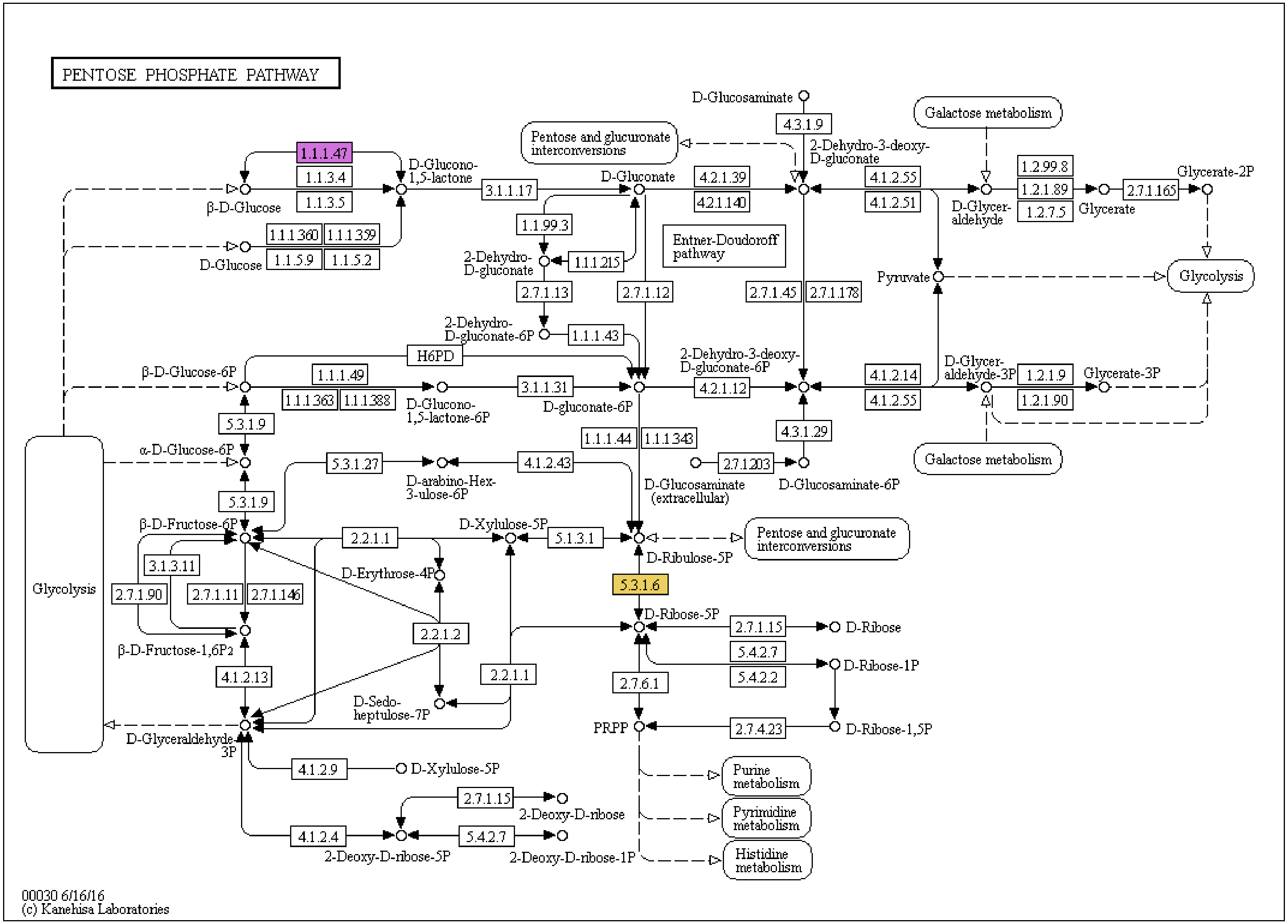

Supplementary Figure S3: KEGG map of Pentose Phosphate pathway[1–4]

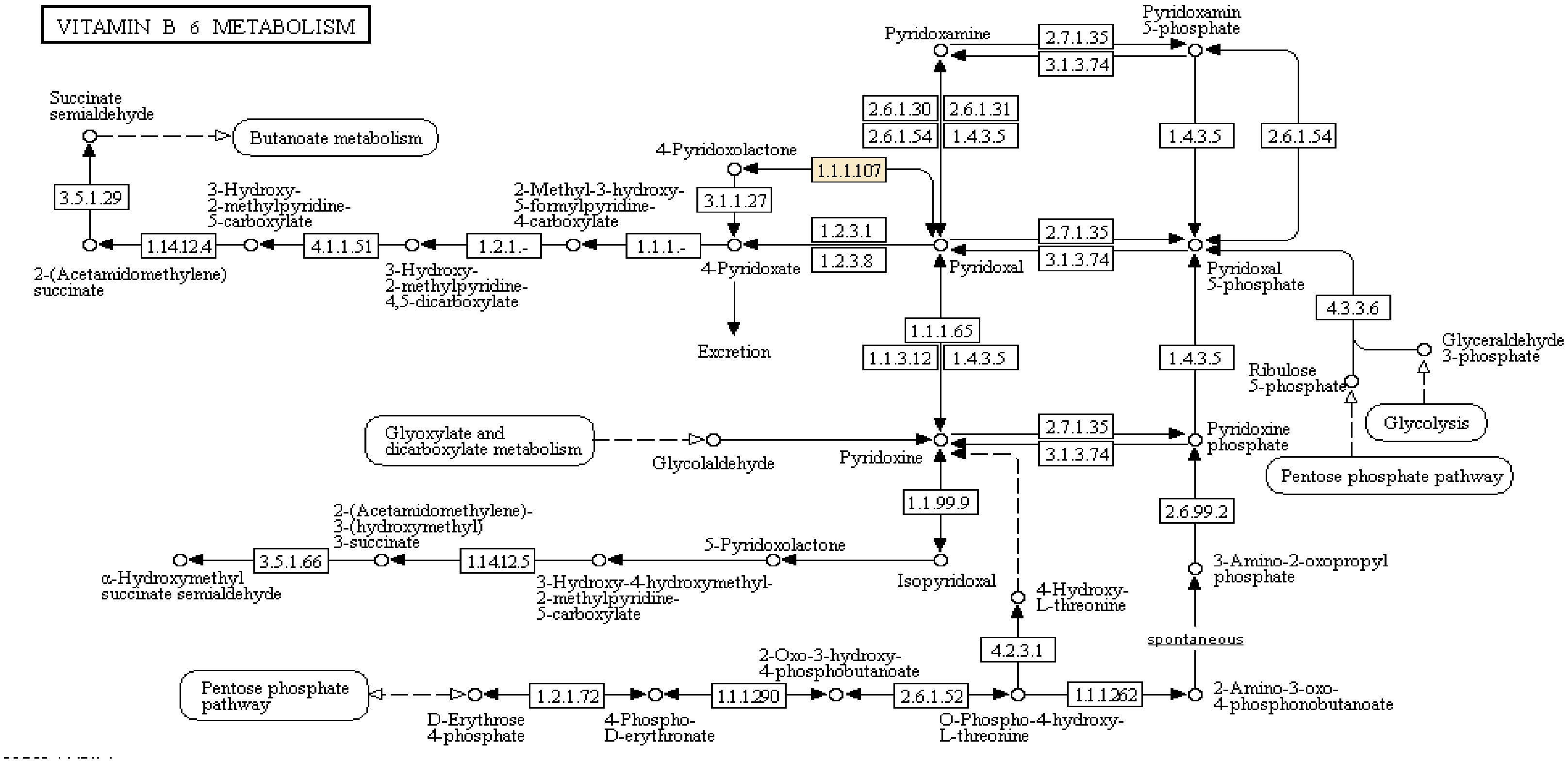

Supplementary Figure S4: KEGG map of Vitamin B6 metabolism pathway[1–4]

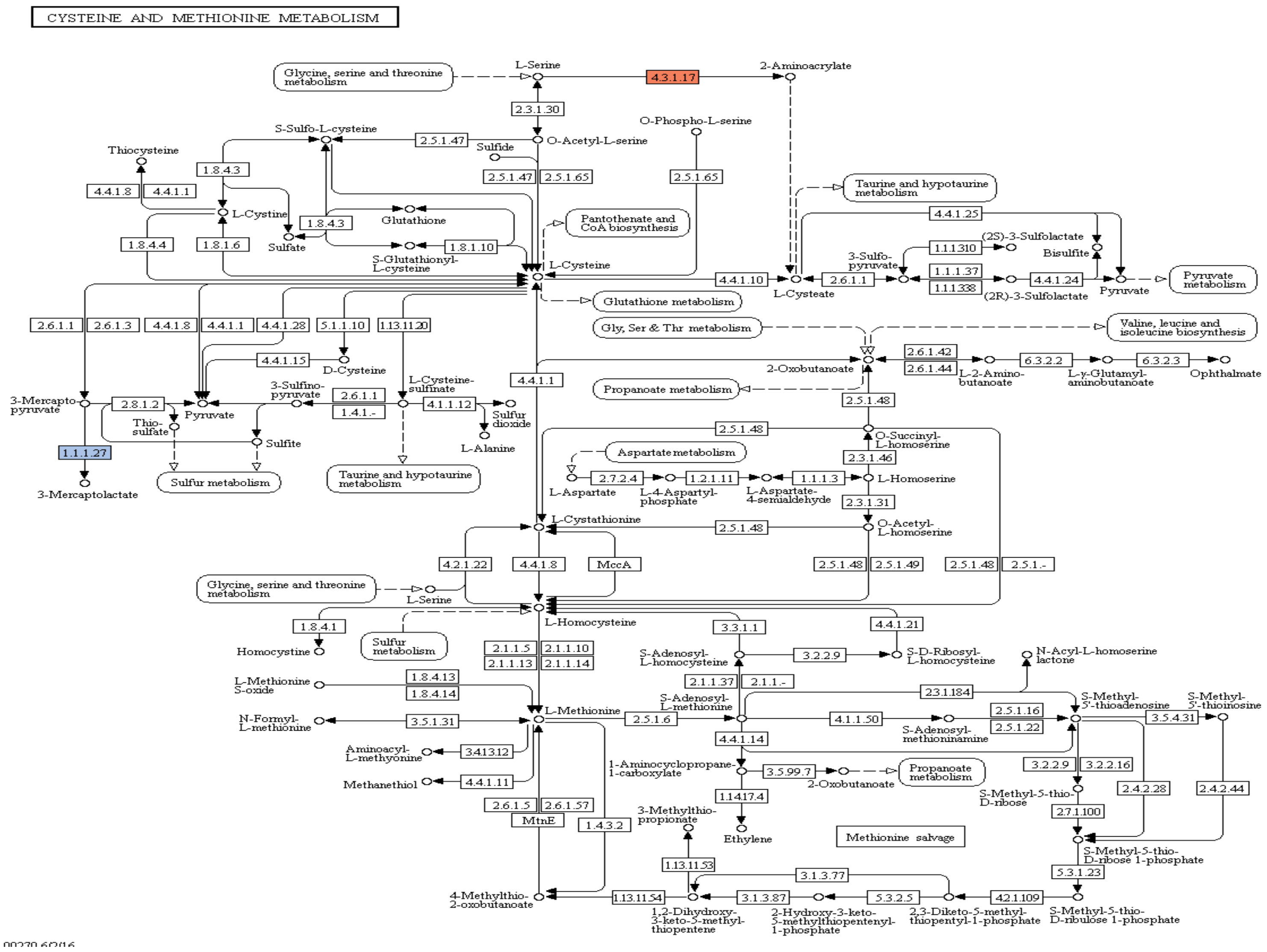

Supplementary Figure S5: KEGG map of cysteine and methionine metabolism pathway[1–4]

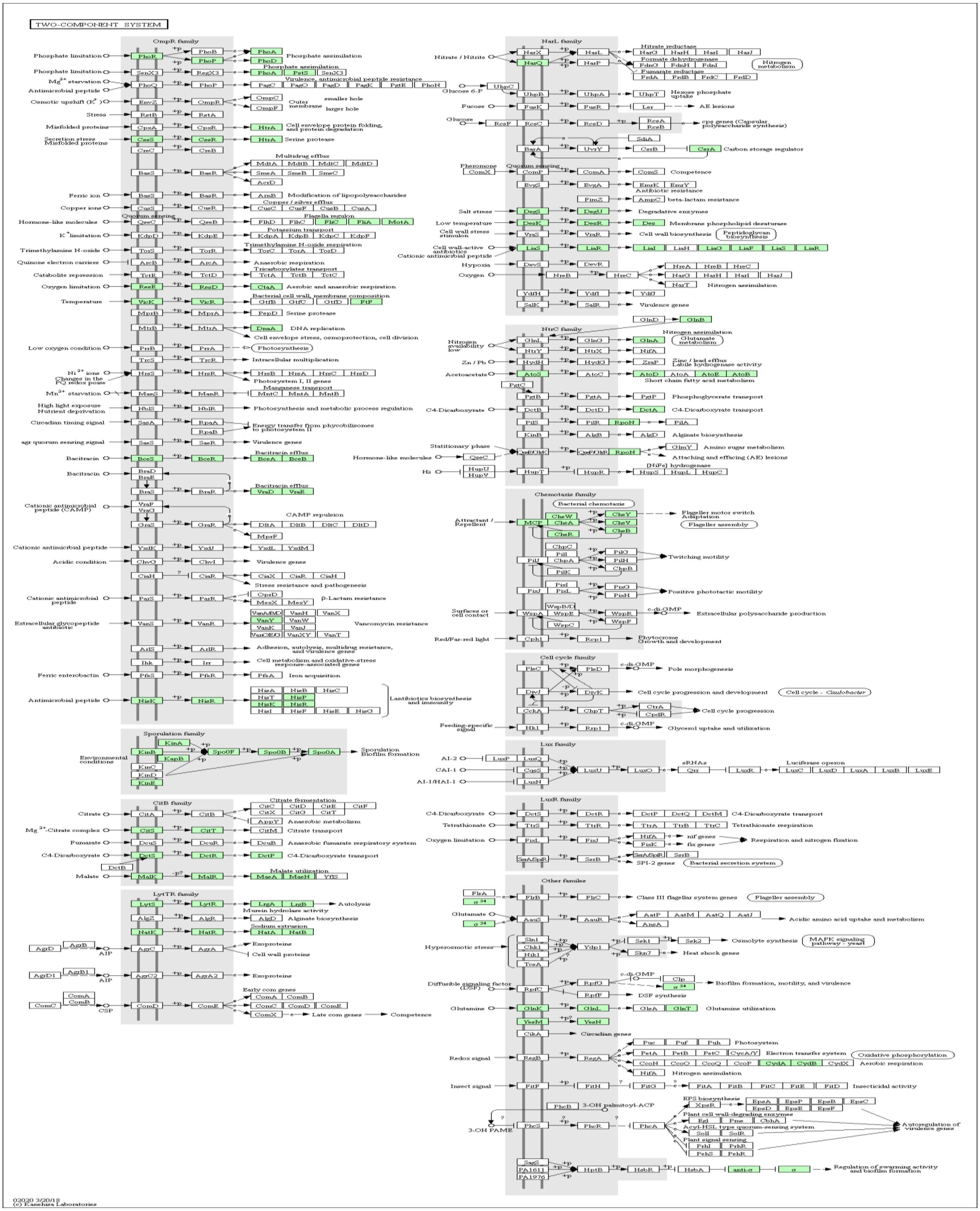

Supplementary Figure S6: KEGG map of the two-component system[1–4]

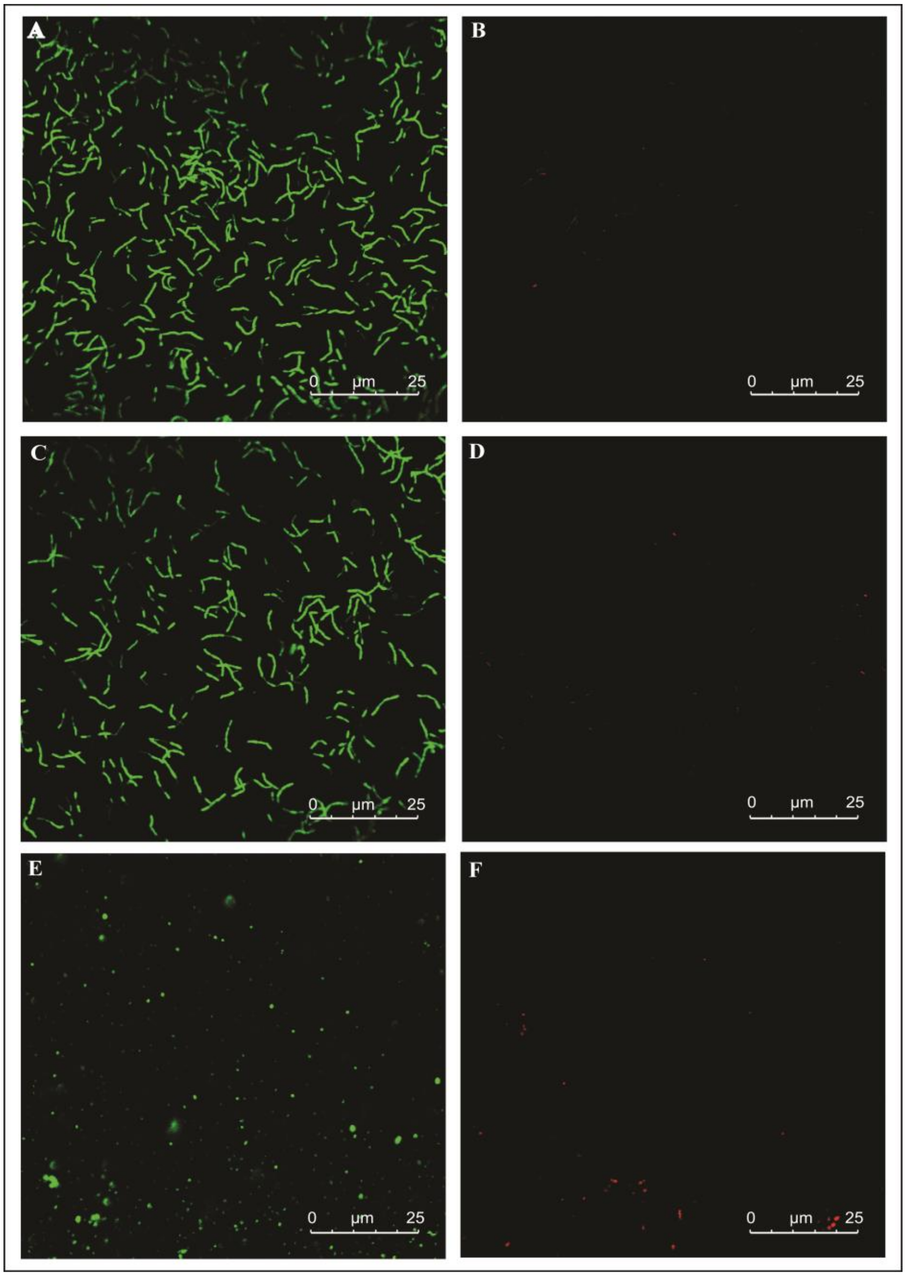

Supplementary Figure S7: Representative results of Live/Dead BacLight staining on *Bacillus megaterium* G18 cells and spores. The cells and spores were incubated for 5 min in the presence of 1µL of 1.67 mM propidium iodide (PI) and 1.67 mM SYTO 9. Image of bacterial cells at 500nm (green) for SYTO9 signal (A) and at 635nm (red) for PI signal (B) grown at pH 7.0 for 4h; image of bacterial cells at 500nm (green) for SYTO9 signal (C) and at 490/635nm (red) for PI signal (D) grown at pH 4.5 for 4h; image of bacterial spores at 500nm (green) for SYTO9 signal (E) and at 635nm (red) for PI signal (F). (Magnification: 10X for all pictures)

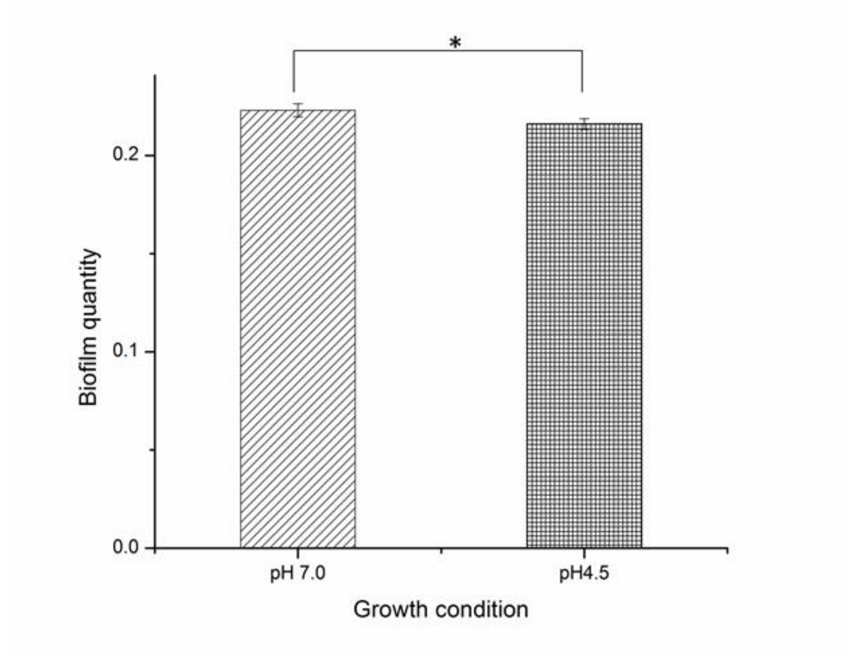

Supplementary Figure S8: Biofilm production by *Bacillus megaterium* G18 at pH 7.0 and pH 4.5, error bar represents the standard error of the mean (n=3) and the asterisk (*) above the bars indicates significant difference ($p < 0.01$).

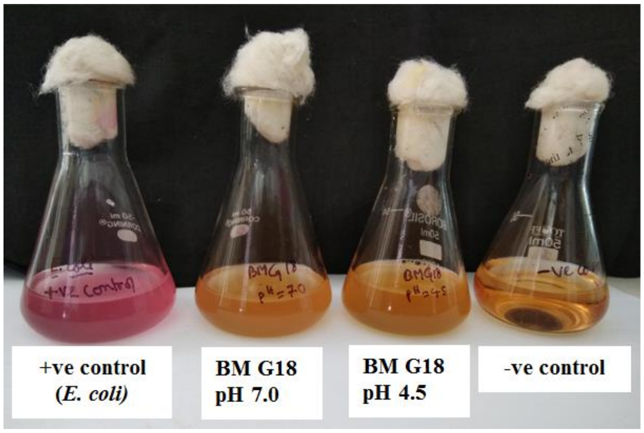

Supplementary Figure S9: Urease was not produced by *B. megaterium* G18 (BM G18: *Bacillus megaterium* G18)

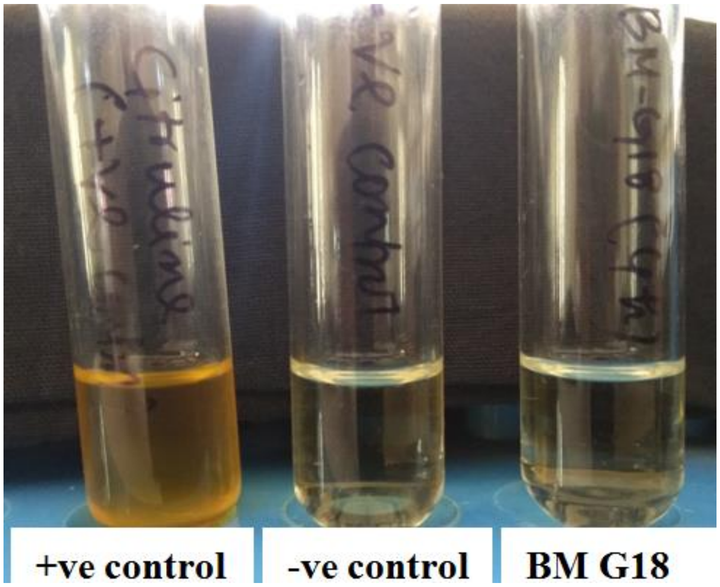

Supplementary Figure S10: Arginine deiminase was not produced by *B. megaterium* G18 under acid stress (BM G18: *Bacillus megaterium* G18)

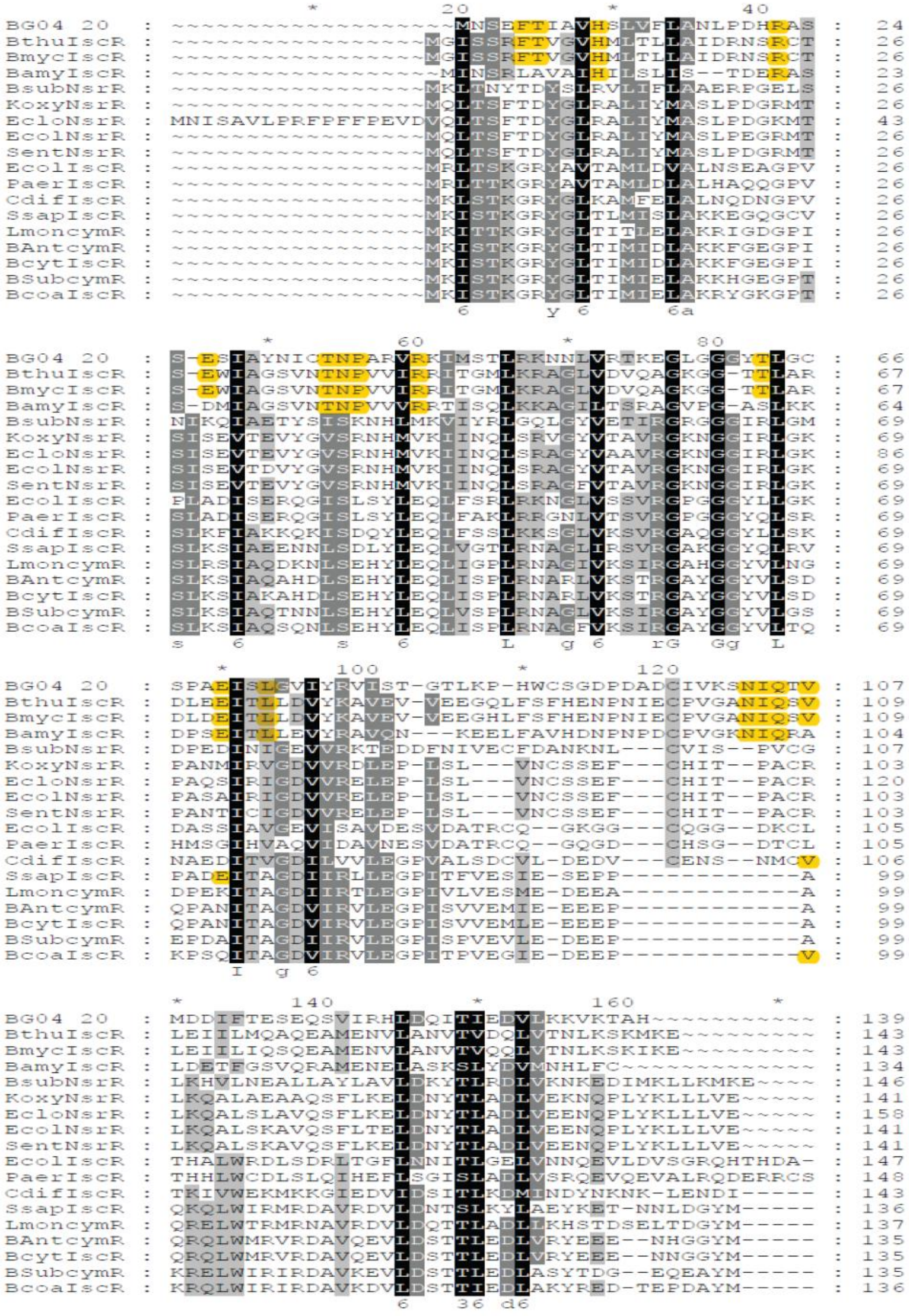

Supplementary Figure S11: Amino acid alignment of *B. megaterium* Rrf2 protein with related proteins. The other Rrf2 proteins shown here include the IscRs from *B. thuringenesis* (BthuIscR), *B. mycoides* (BmycIscR), *B. amyloliquefaciens* (BamyIscR), *E. coli* (EcoIscR), *P. aerugunosa* (PaerIscR), *C. difficle* (CdifIscR), *S. saprophyticus* (SsapIscR), *B. cytotoxicus* (BcytIscR), and *B. coagulans* (BcoaIscR); NsrRs from *B. subtilis* (BsubNsrR), *K. oxytoca* (KoxyNsrR), *E. cloacae* (EcloNsrR), *E. coli* (EcNsrR), and *Salmonella enterica* (SentNsrR); and cymRs from *B. subtilis*

(BsubcymR), *B. anthracis* (BAntcymR) and *L. monocytogens* (LmoncymR). The alignment was carried out using Clustal Omega (https://www.ebi.ac.uk/Tools/msa/clustalo) and presented using Genedoc (http://genedoc.software.informer.com/2.7/). Secondary structural elements of the Rrf2 protein are indicated above the sequences. Black colour indicates totally conserved residues, grey indicates highly conserved residues, and light grey indicates residues that are either well conserved or conservatively substituted. Yellow colour indicates the similar residues present in the IscR from *B. thuringenesis* (BthuIscR), *B. mycoides* (BmycIscR), and *B. amyloliquefaciens* (BamyIscR).

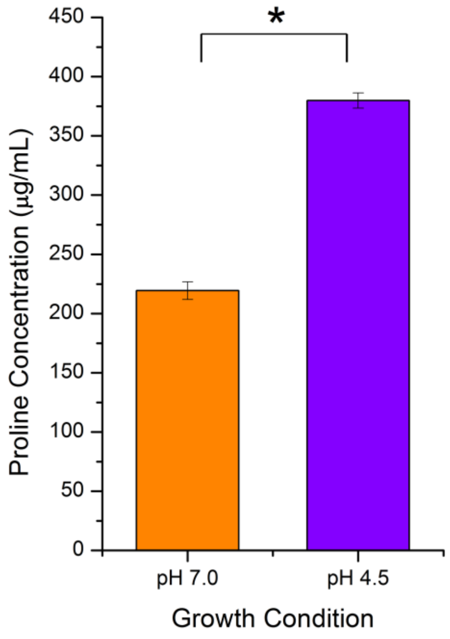

Supplementary Figure S12: Intracellular proline content in *B. megaterium* G18.
The log phase culture (OD_{600} = 1.0) of *B. megaterium* G18 was shifted to pH 7.0 and pH 4.5 and allowed to grow for 4h and after which intracellular proline content was measured. The error bar represents the standard error of the mean (n=3) and the asterisk (*) above the bars indicates significant difference ($p < 0.01$).

Reference:

1. Kanehisa, M. & Goto, S. KEGG: kyoto encyclopedia of genes and genomes. *Nucleic Acids Res.* **28,** 27–30 (2000).
2. Moriya, Y., Itoh, M., Okuda, S., Yoshizawa, A. C. & Kanehisa, M. KAAS: an automatic

genome annotation and pathway reconstruction server. *Nucleic Acids Res.* **35,** W182–W185 (2007).

3. Kanehisa, M., Sato, Y., Kawashima, M., Furumichi, M. & Tanabe, M. KEGG as a reference resource for gene and protein annotation. *Nucleic Acids Res.* **44,** D457-62 (2016).

4. Kanehisa, M., Furumichi, M., Tanabe, M., Sato, Y. & Morishima, K. KEGG: new perspectives on genomes, pathways, diseases and drugs. *Nucleic Acids Res.* **45,** D353–D361 (2017).
